# Supplementary figures and images for: A multi-scale cortical wiring space links cellular architecture and functional dynamics in the human brain
Source: PLoS Biol. 2020 Nov 30;18(11):e3000979. doi: 10.1371/journal.pbio.3000979 (PMC7728398; doi:10.1371/journal.pbio.3000979)

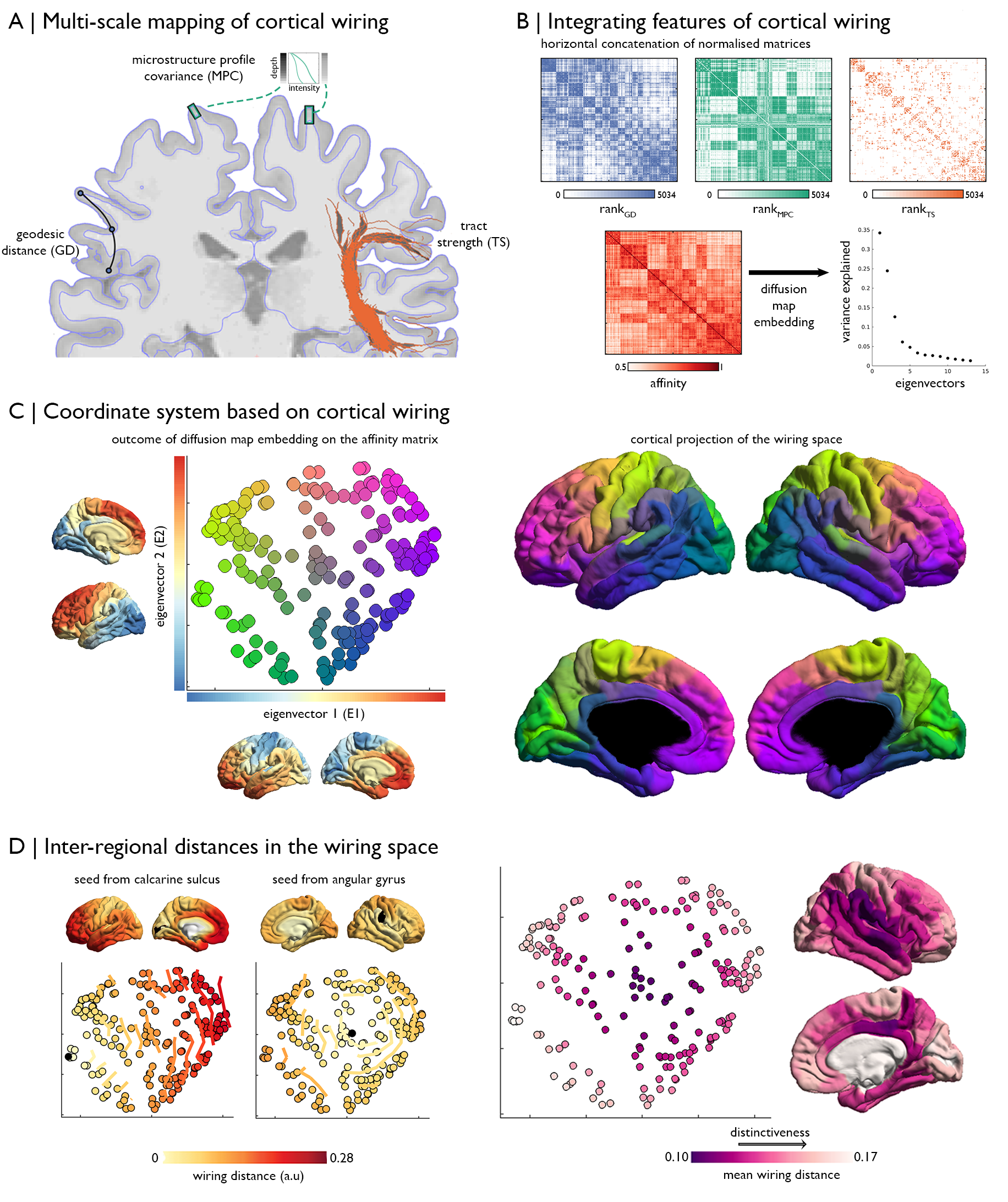

Supplement: S1 Fig — (A) Wiring features, i.e., GD, MPC, and diffusion-based TS were estimated between all pairs of nodes. (B) Normalised matrices were concatenated and transformed into an affinity matrix. Manifold learning identified a lower-dimensional space determined by cortical wiring. (C) Left: node positions in this newly discovered space, coloured according to proximity to axis limits. Closeness to the maximum of the second eigenvector is redness, towards the minimum of the first eigenvector is greenness, and towards the maximum of the first eigenvector is blueness. The first 2 eigenvectors are shown on the respective axes. Right: Equivalent cortical surface representation. (D) Calculation of interregional distances (isocontour lines) in the wiring space from specific seeds to other regions of cortex (left). Overall distance to all other nodes can also be quantified to index centrality of different regions, with more integrative areas having shorter distances to nodes (right). Essential data are available on https://git.io/JTg1l. a.u., arbitrary units; GD, geodesic distance; MPC, microstructure profile covariance; TS, tractography strength. (TIF) [file pbio.3000979.s001.tif]

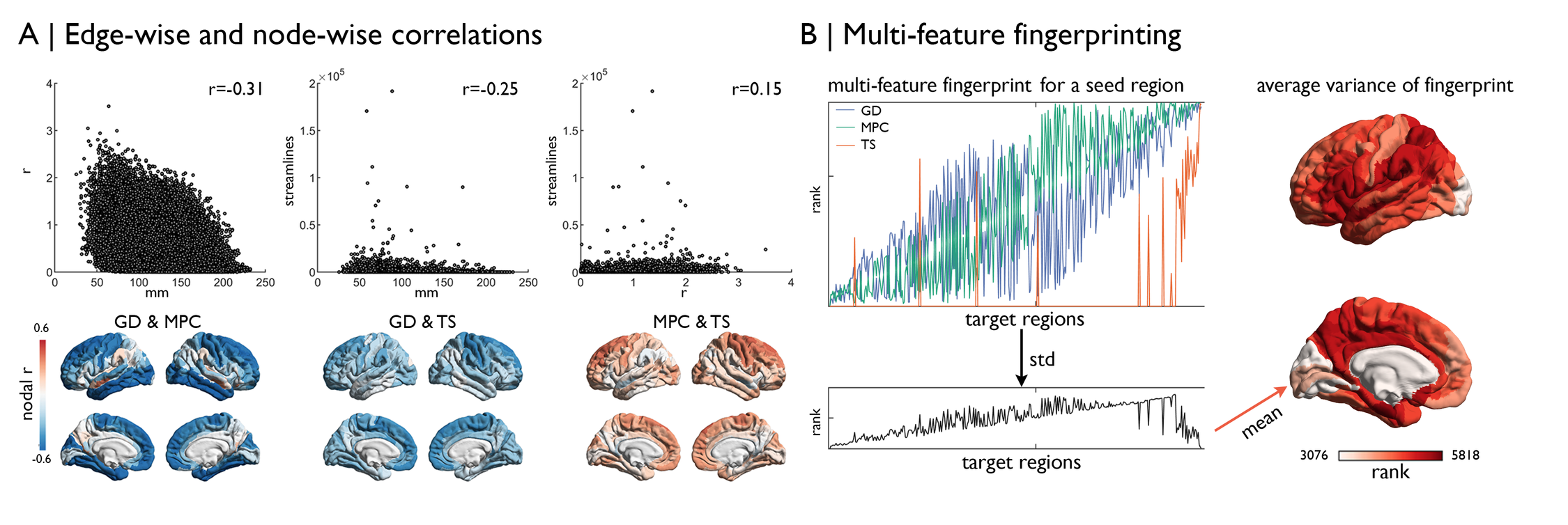

Supplement: S2 Fig — (A) Scatterplots depict the global correlation between the different cortical wiring features, i.e., GD, MPC, and diffusion-based TS. Average r values for each node are projected below on the cortical surface. (B) Fingerprinting involved combining all 3 wiring features of 1 region. The SD across the 3 features was estimated for each edge, then the average was taken for each node as a measure of feature variance of the fingerprint. Essential data are available on https://git.io/JTg1l. GD, geodesic distance; MPC, microstructure profile covariance; SD, standard deviation; TS, tractography strength. (TIF) [file pbio.3000979.s002.tif]

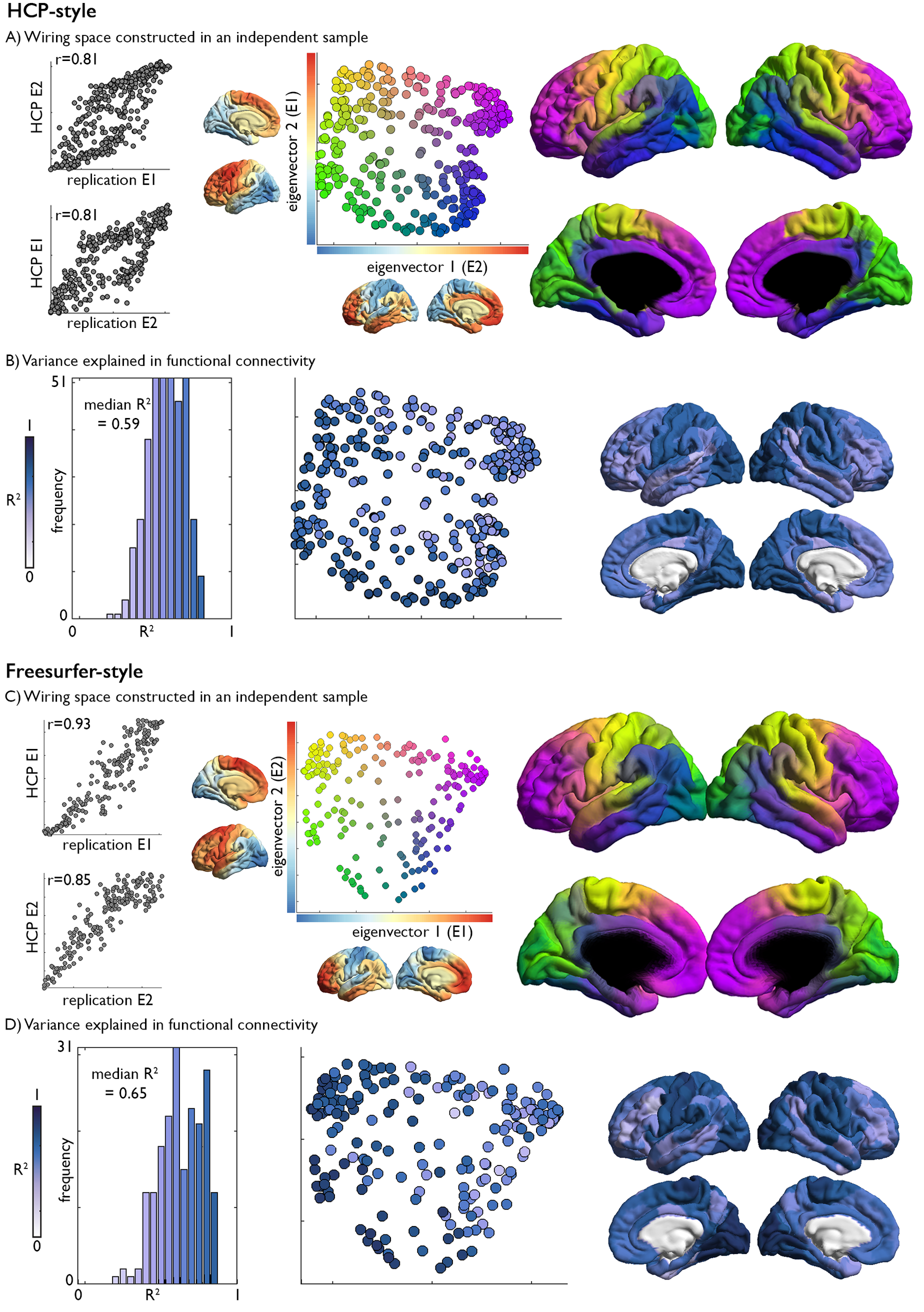

Supplement: S3 Fig — The wiring space was regenerated using an independent MICs dataset of 40 healthy adults scanned at our imaging centre. Imaging parameters were similar to the original cohort, albeit using quantitative T1 relaxometry as a marker of cortical microstructure rather than HCP’s T1w/T2w ratio mapping. (A|C) Scatterplots show marked correspondence between the original HCP “Discovery” sample eigenvectors and those from the MICs dataset, with Spearman correlations shown. (B|D) Using boosting regression, we similarly found that high, but regionally variable, variance in the group average functional connectivity could be explained by the wiring space in the MICs dataset. Essential data are available on https://git.io/JTg1l. HCP, Human Connectome Project; MICs, Microstructure Informed Connectomics. (TIF) [file pbio.3000979.s003.tif]

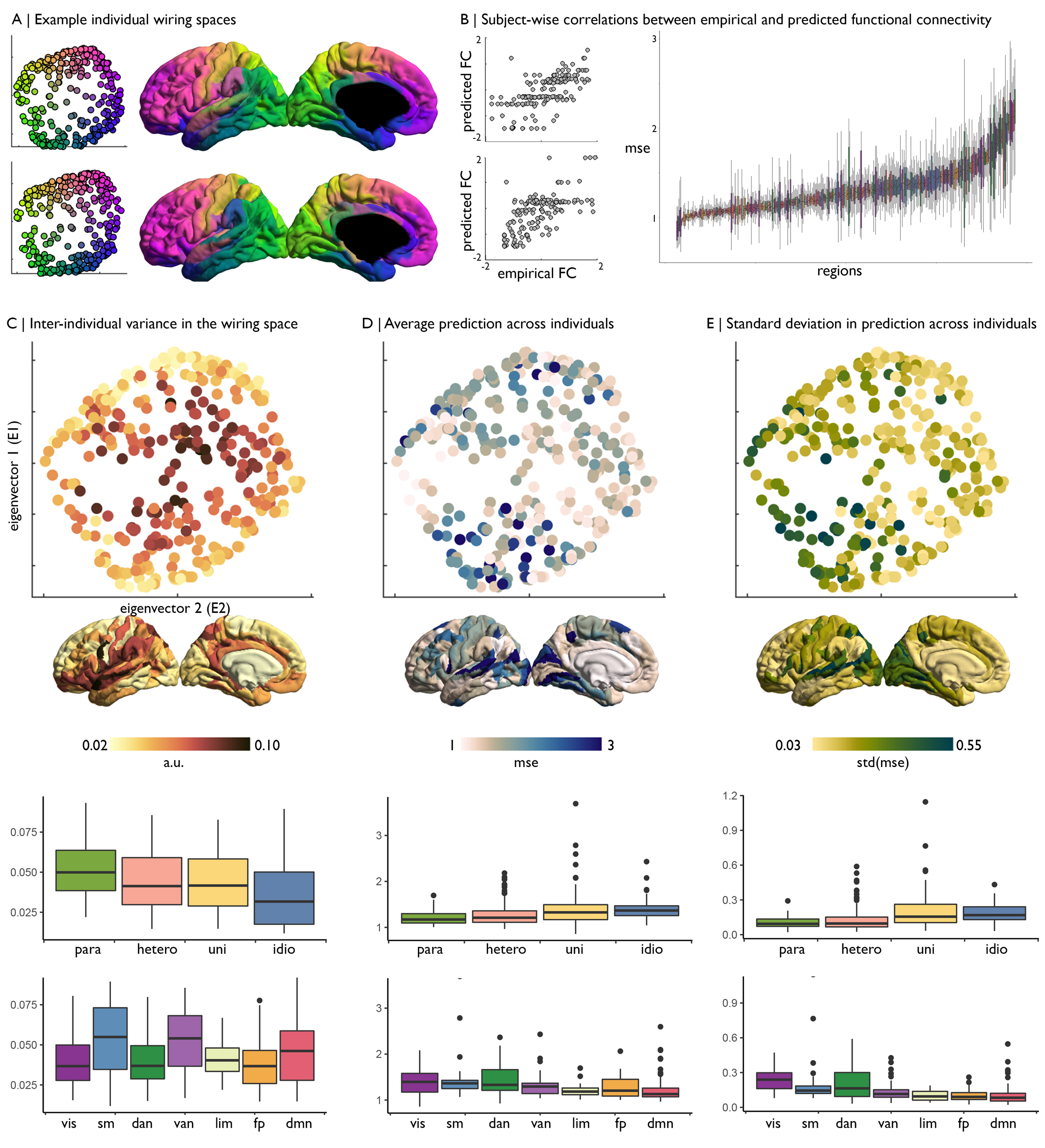

Supplement: S4 Fig — (A) Wiring spaces constructed in 2 individuals of the “Hold-out,” which were aligned to the group-level “Discovery” wiring space. (B) MSE between predicted and empirical functional connectivity. Each box represents a node, and the distribution is taken across individuals. Box are coloured by the assignment of the node to a functional community (Fig 3). (C–E) Individual variation was calculated as the Euclidean distance between the node’s position in the subject vs group average wiring space, where the position is synonymous with the values on the first 2 eigenvectors following Procrustes alignment. Interindividual variance was taken as the average across all participants and provided as a.u. We also calculated the mean and SD in the MSE between predicted and empirical functional connectivity for each node across participants. Node-wise values are presented in the wiring space, on the cortical surface and stratified by level of laminar differentiation [7, 133] and functional network [49]. Essential data are available on https://git.io/JTg1l. a.u., arbitrary units; MSE, mean squared error; rs-fMRI, resting-state functional magnetic resonance imaging; SD, standard deviation. (TIF) [file pbio.3000979.s004.tif]

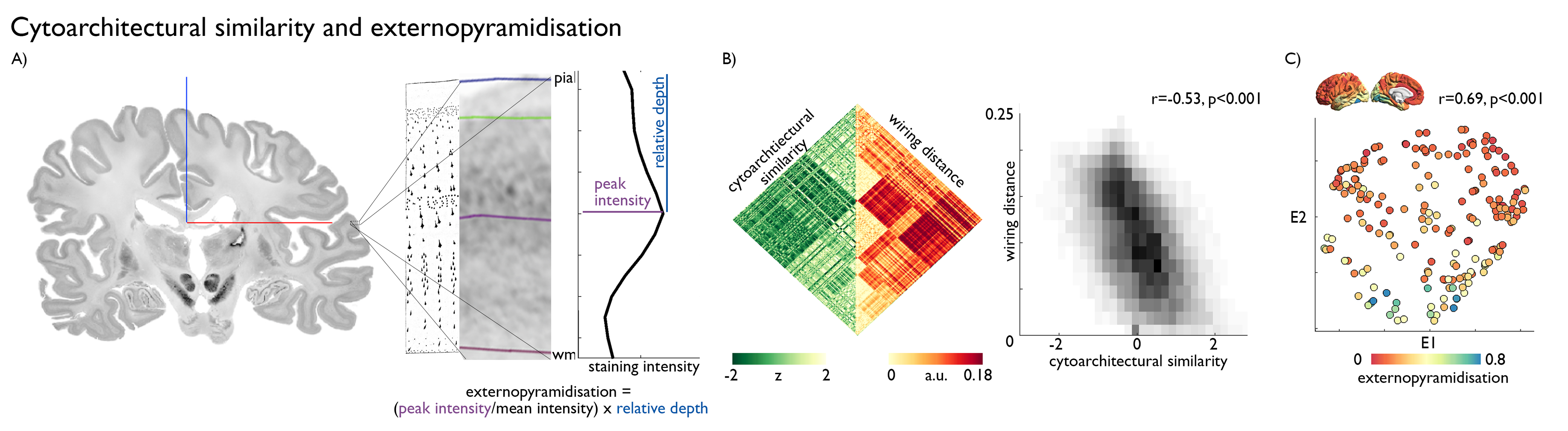

Supplement: S5 Fig — (A) A 3D postmortem histological reconstruction of a human brain [35] was used to estimate cytoarchitectural similarity and externopyramidisation. Here, we present a coronal slice, a drawing of cytoarchitecture, a magnified view of cortical layers in BigBrain, and a staining intensity profile with example of calculation of externopyramidisation [44]. (B) Matrix and density plot depict the correlation between BigBrain-derived cytoarchitectural similarity and wiring distance between pairs of regions. (C) Externopyramidisation, projected onto the cortical surface and into the wiring space, is highest at the bottom of the structural manifold. Essential data are available on https://git.io/JTg1l. (TIF) [file pbio.3000979.s005.tif]

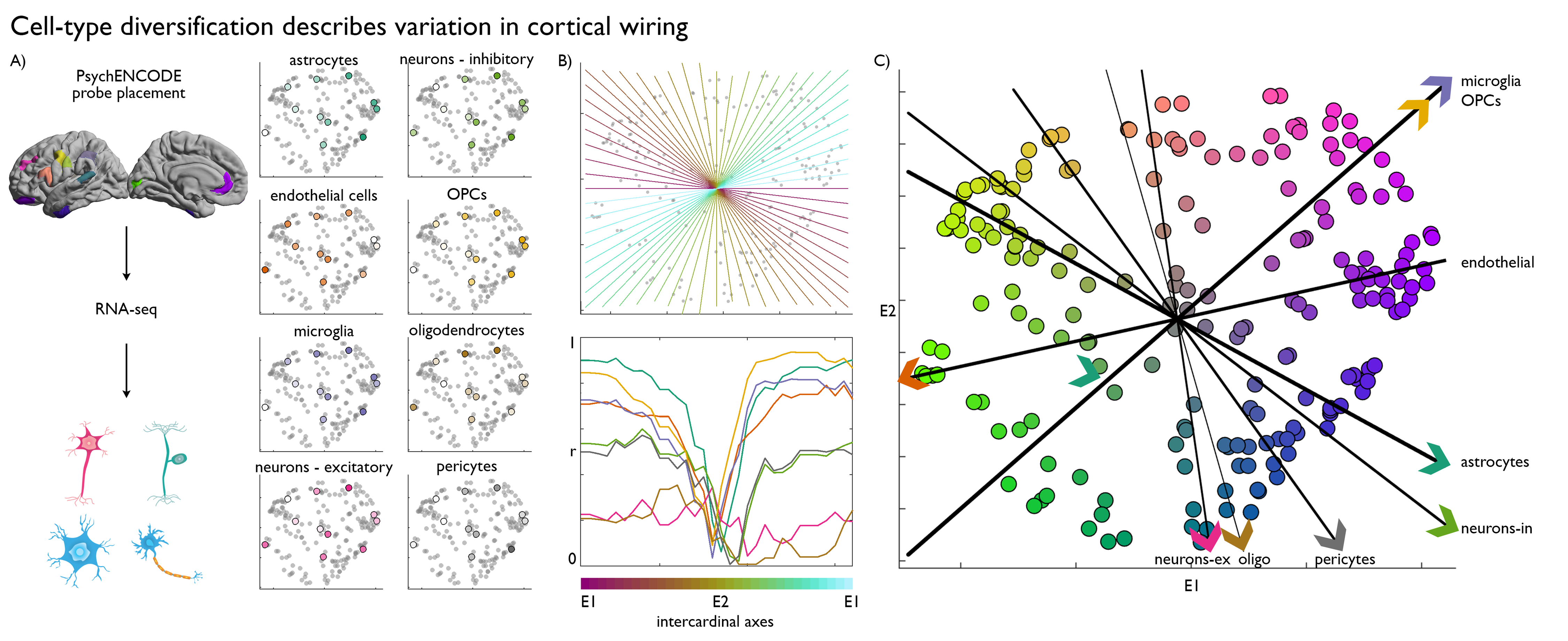

Supplement: S6 Fig — (A) mRNA-seq probes, assigned to 11 representative nodes (coloured as in Fig 1C, i.e., their position in the wiring space), provided good coverage of the space, and enabled characterisation of cell type–specific gene expression patterns. Average cell type–specific gene expression patterns projected in the wiring space, with brighter colours signifying higher expression. (B) Equally spaced intercardinal axes superimposed on the wiring space, and below, line plots showing correlation of gene expression patterns with each of the axes. Colours correspond to the cell types shown in part A. (C) Strongest axis of variation (i.e., maximum |r|) in expression of each cell type overlaid on the structural manifold. Essential data are available on https://git.io/JTg1l. mRNA-seq, mRNA-sequencing. (TIF) [file pbio.3000979.s006.tif]

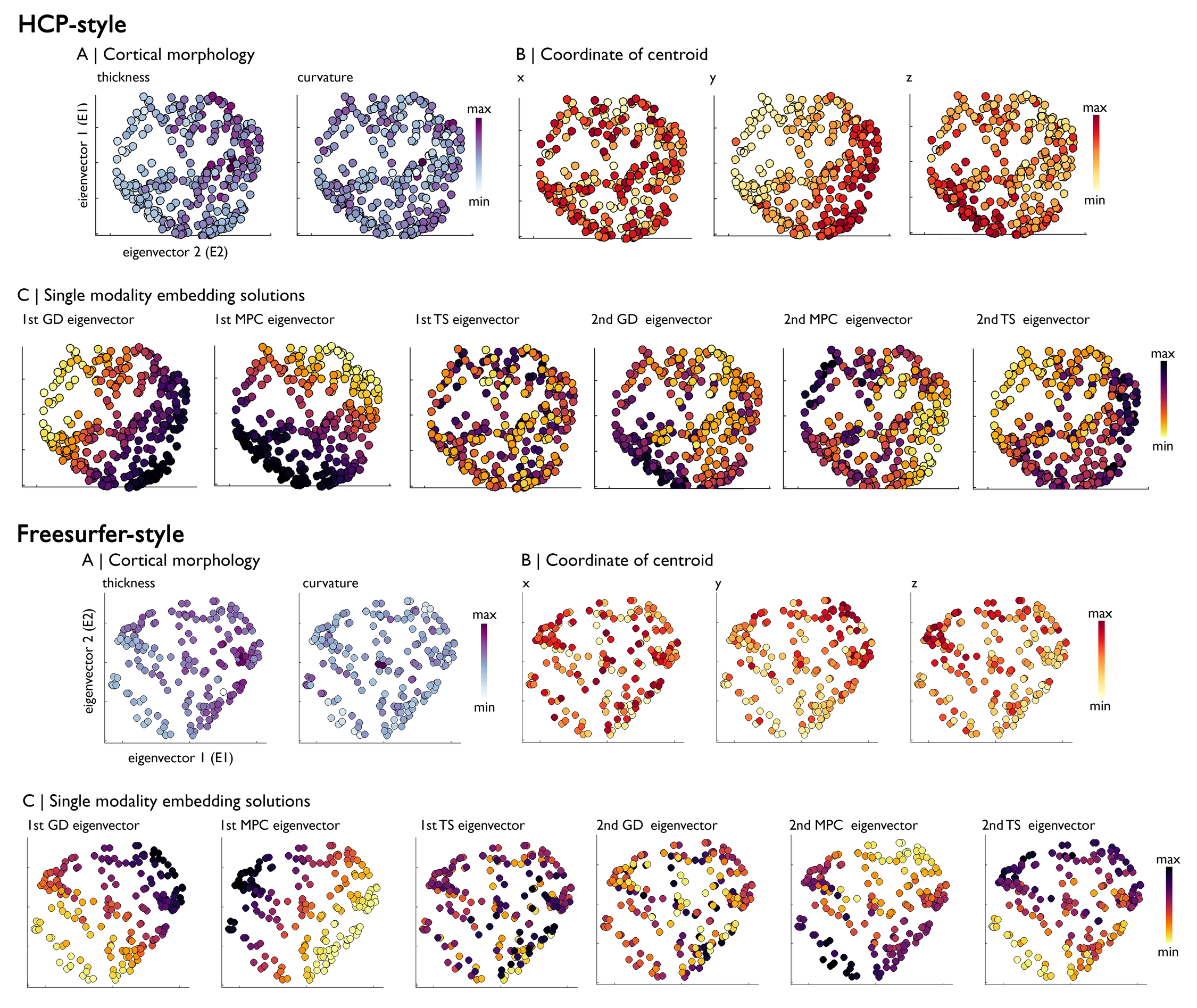

Supplement: S7 Fig — (A) Group average cortical thickness and curvature measures in the manifold. (B) Centroid coordinate of each parcel. x = left–right, y = posterior–anterior, z = inferior–superior. (C|D) Principles gradients from single modalities. Essential data are available on https://git.io/JTg1l. (TIF) [file pbio.3000979.s007.tif]

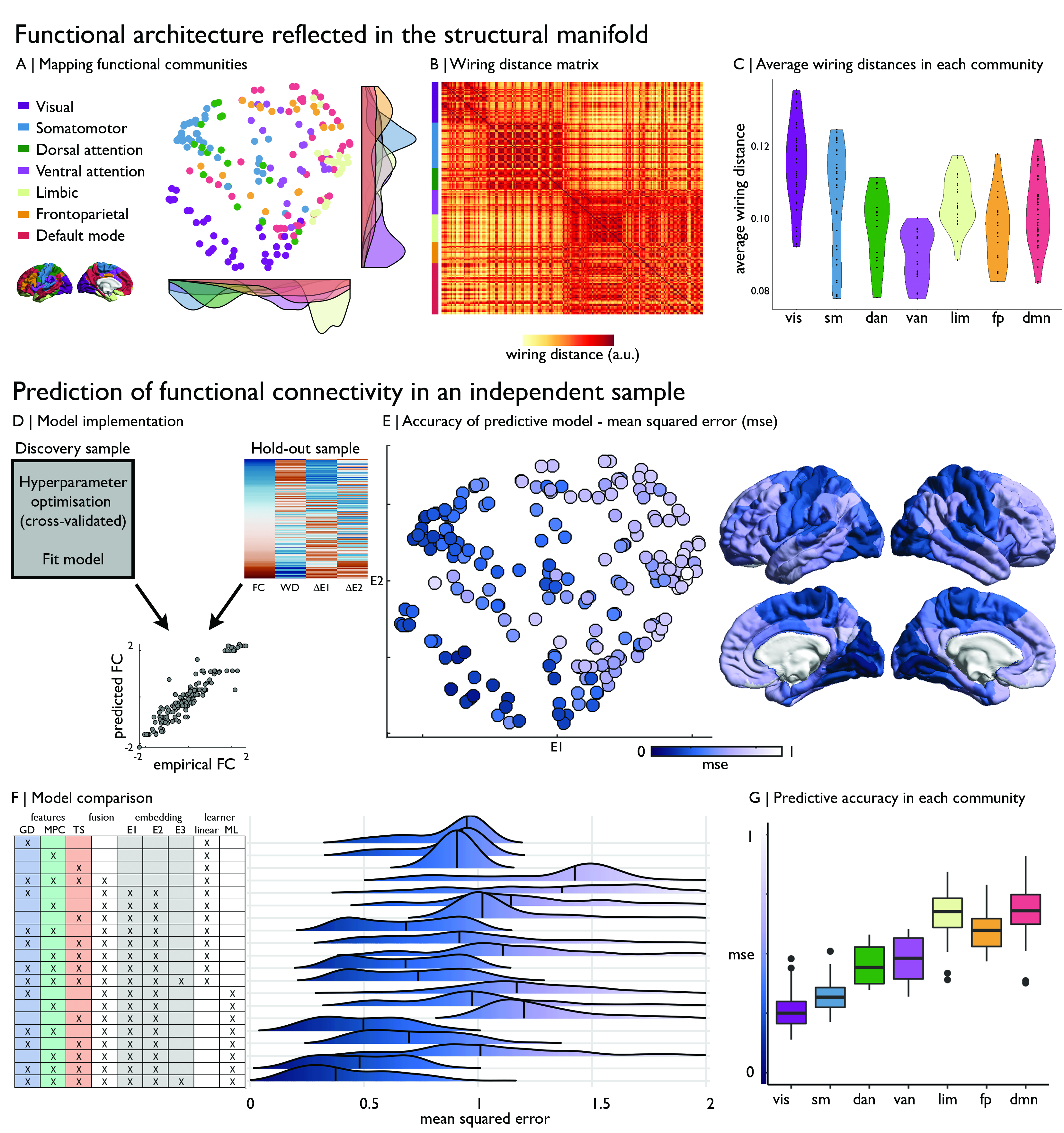

Supplement: S8 Fig — (A) Nodes in the wiring-derived coordinate system coloured by functional community [47], with the distribution of networks shown by density plots along the axes. (B) Wiring distance between nodes, ordered by functional community, revealed a modular architecture. (C) Violin plots show the average wiring distance for nodes in each functional community, with higher values being more specialised in their cortical wiring. (D) Using the boosting regression models from the “Discovery” dataset, we used features of the wiring space to predict z-standardised functional connectivity in a “Hold-out” sample. The model was enacted for each node separately. (E) MSE across nodes are shown in the wiring space and on the cortical surface (S3 Fig). (F) Predictive accuracy of various cortical wiring models, involving the use of different features, multifeature fusion, eigenvectors from diffusion map embedding, and a linear or ML learner. (G) MSE of the wiring space model stratified by functional community. Essential data are available on https://git.io/JTg1l. ΔE1, difference on eigenvector 1; ΔE2, difference on eigenvector 2; FC, functional connectivity; ML, machine learning; MSE, mean squared error; WD, wiring distance. (TIF) [file pbio.3000979.s008.tif]

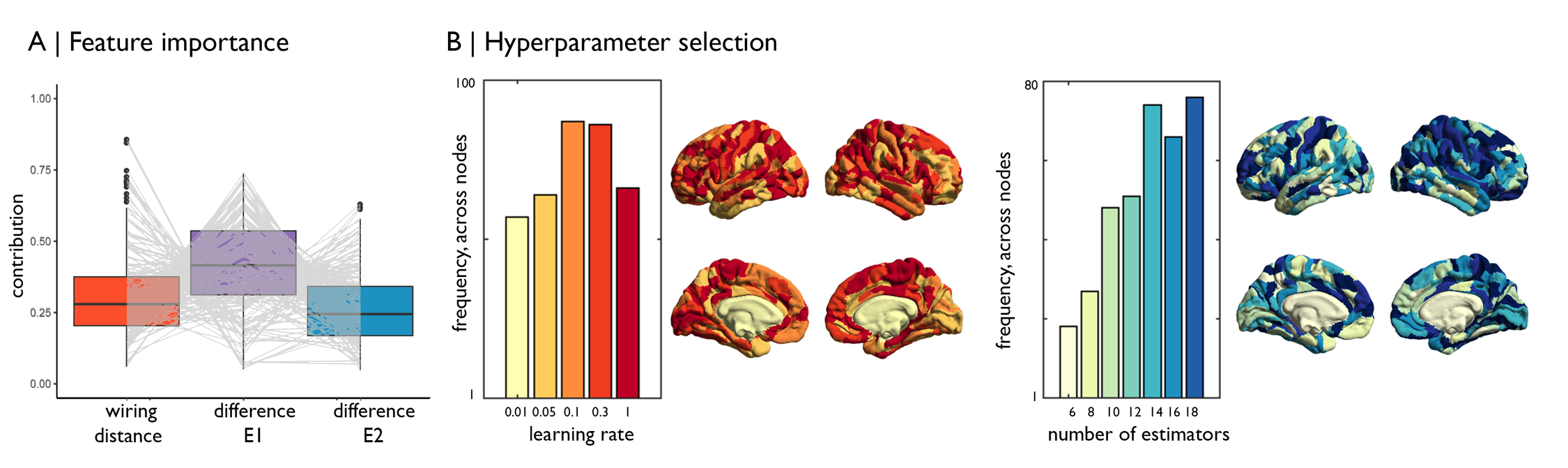

Supplement: S9 Fig — (A) Box and spaghetti plots depict the relative importance of wiring space features for the boosting regression model at each node. (B) Learning rate and number of estimated selected by cross-validation for the model at each node. Essential data are available on https://git.io/JTg1l. fMRI, functional magnetic resonance imaging. (TIF) [file pbio.3000979.s009.tif]

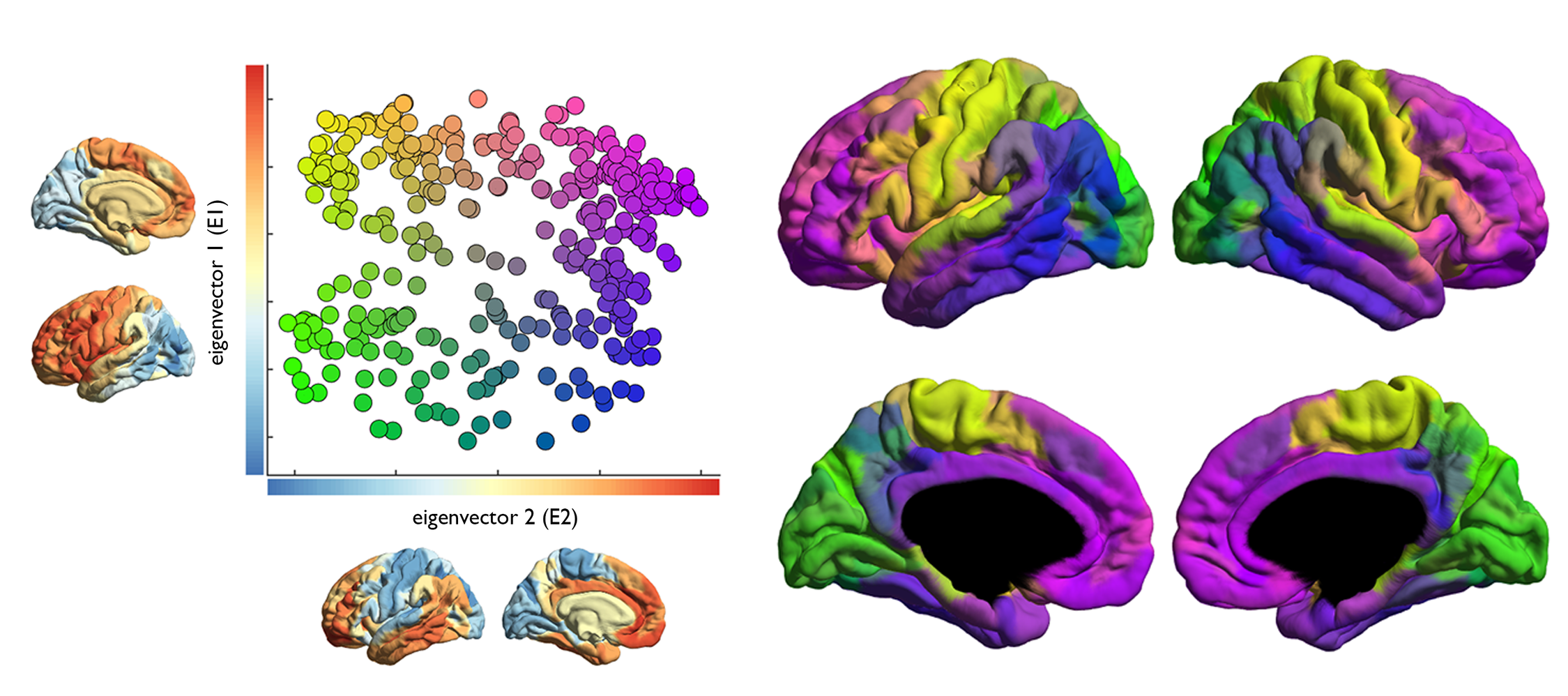

Supplement: S10 Fig — The wiring space generated from the group average of cortical wiring features across the patients was highly similar to the healthy template (correlations between eigenvectors 1/2: r = 0.92/0.89). Essential data are available on https://git.io/JTg1l. (TIF) [file pbio.3000979.s010.tif]

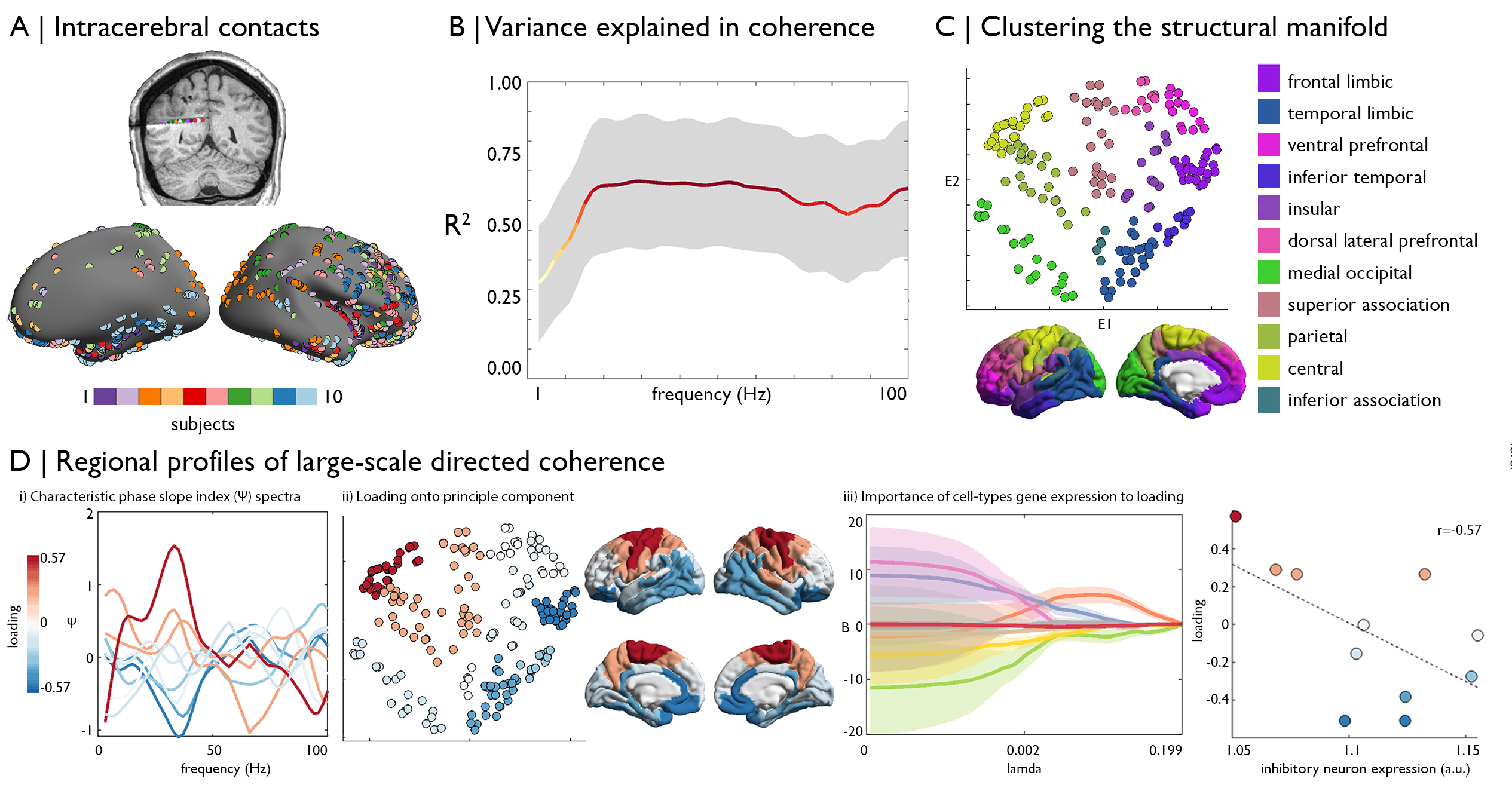

Supplement: S11 Fig — (A) Intracerebral implantations of 10 epileptic patients were mapped to the cortical surface and intracortical EEG contacts selected. We studied 5 minutes of wakeful rest. (B) Mean and SD in the variance explained in undirected coherence by wiring space features using adaboost machine learning across all nodes. (C) Clusters of the wiring space. (D) Phase slope index (Ψ) was calculated for each pair of intra-subject electrodes, then cluster-to-cluster estimates were derived from a linear mixed-effect model. Pearson correlation across Ψ estimates was used to measure the similarity of clusters, and the major axis of regional variation was identified via principle component analysis. (i) Average Ψ spectra for each region coloured by loading on the first principle component (accounting for 39% of variance). (ii) Component loadings presented in the wiring space and on the cortical surface illustrate a gradient from upper left regions, corresponding to central areas, towards lower right areas, corresponding to temporal and limbic areas. (iii) Lasso regularisation demonstrates the contribution of cell type–specific gene expression (colours matching Fig 3) and externopyramidisation (red) to explain variance in the component loadings. Shaded areas show the SD in fitted least-squares regression coefficients across leave-one-observation-out iterations. For example, inhibitory neurons expression levels (green) are closely related to the component loading, as shown in the scatterplot. Essential data are available on https://git.io/JTg1l. EEG, electroencephalography; SD, standard deviation. (TIF) [file pbio.3000979.s011.tif]

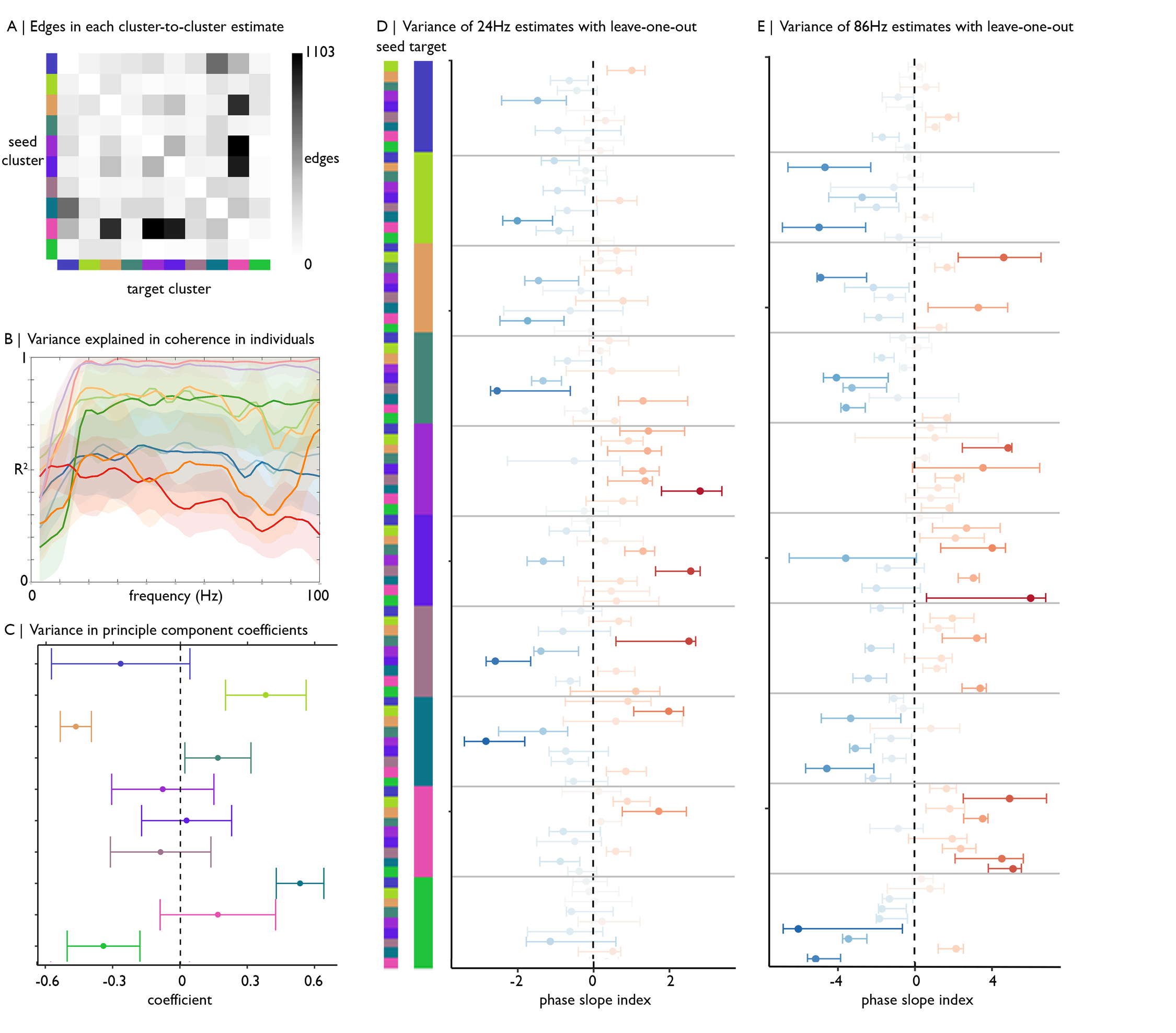

Supplement: S12 Fig — (A) Matrix depicting the number of intra-subject electrode pairs that contribute to the cluster-to-cluster estimations of phase slope index. (B) Variance explained in coherence by boosting regression, operationalised at a single subject level with individualised wiring spaces. (C) Variance in coefficients from a principle component analysis of intercluster similarities in the phase slope index using a leave-one-subject-out procedure. (D) and (E) involve the same leave-one-subject-out procedure to gauge the variance in edge-wise phase slope index estimates at frequencies of interest. Notably, the range of estimates does not pass 0 for significant edges. Essential data are available on https://git.io/JTg1l. EEG, electroencephalography. (TIF) [file pbio.3000979.s012.tif]

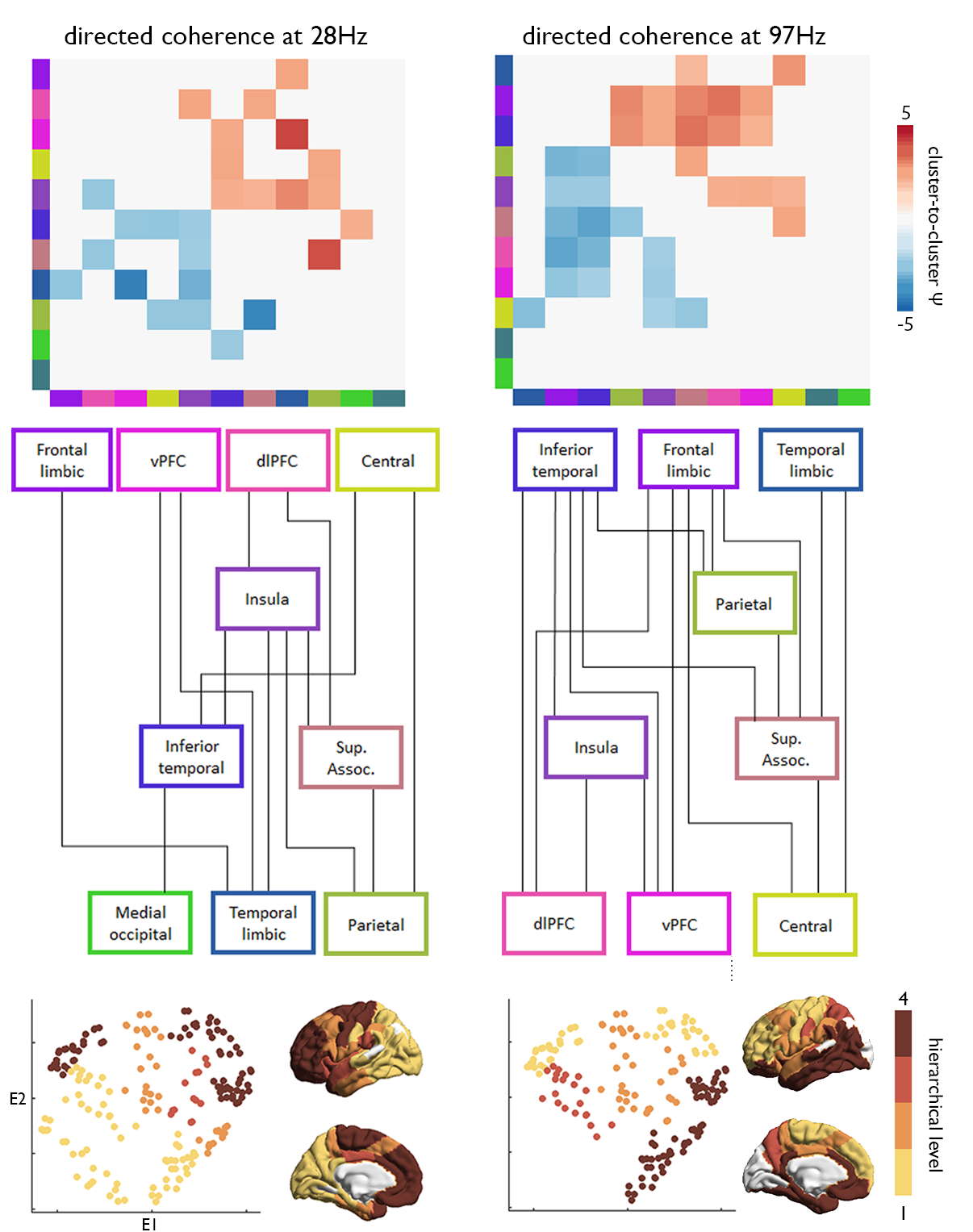

Supplement: S13 Fig — “Top”: The most influential frequencies on the component loading were identified through a correlation of average Ψ spectra (Fig 5Di) with the component loading. For the global maxima (28 Hz, left) and minima (97 Hz, right), we present cluster-to-cluster Ψ estimates, thresholded at p < 0.05. “Bottom”: Suprathreshold edges are plotted as a hierarchical schema, where the directed coherence estimates indicate flow of oscillations from the top to the bottom of the hierarchy. The hierarchical level of each cluster is also presented on the cortical surface and in the wiring space, illustrating unique spatial patterns of the frequency-specific hierarchies. Compared to the HCP-style pipeline, the frequencies of interest are slightly shifted resulting in distinct hierarchies. Essential data are available on https://git.io/JTg1l. HCP, Human Connectome Project. (TIF) [file pbio.3000979.s013.tif]
