# Supplementary material for: A multi-scale cortical wiring space links cellular architecture and functional dynamics in the human brain
Source: PLoS Biol. 2020 Nov 30;18(11):e3000979. doi: 10.1371/journal.pbio.3000979 (PMC7728398; doi:10.1371/journal.pbio.3000979)
Supplement: S1 Text — (DOCX) [file pbio.3000979.s014.docx]

**S1 Text: A multi-scale cortical wiring space links cellular architecture and functional dynamics in the human brain**

**Table A**: Variance explained (R^2^) in externopyramidisation with alternative procedures and input measures

|  | HCP-style | Freesurfer-style |
| --- | --- | --- |
| GD only | 0.2931 | 0.50975 |
| MPC only | 0.2062 | 0.49381 |
| TS only | 0.2577 | 0.62106 |
| GD + MPC | 0.2579 | 0.64716 |
| GD + TS | 0.2832 | 0.52581 |
| MPC + TS | 0.2239 | 0.54945 |
| GD, MPC + TS | 0.2609 | 0.65076 |

**Table B: Statistical relationships between cell-type specific gene expression and wiring space.**

|  | Regional expression modelled by wiring space eigenvectors 1 & 2^1^ | | | | Spearman correlation between co-expression and wiring distance | | | |
| --- | --- | --- | --- | --- | --- | --- | --- | --- |
|  | HCP-style | | Freesurfer-style | | HCP-style | | Freesurfer-style | |
| Cell-type | r | p^2^ | r | p^2^ | r | p | r | p |
| Astrocyte | 0.86 | 0.033 | 0.91 | 0.002 | -0.44 | <0.001 | -0.74 | <0.001 |
| Endothelial cells | 0.81 | 0.018 | 0.81 | 0.282 | -0.34 | 0.012 | -0.55 | <0.001 |
| Microglia | 0.75 | 0.042 | 0.76 | 0.037 | -0.34 | 0.011 | -0.75 | <0.001 |
| Neurons - excitatory | 0 | 0.169 | 0 | 0.127 | -0.42 | 0.002 | -0.68 | <0.001 |
| Neurons -inhibitory | 0.48 | 0.060 | 0.46 | 0.040 | -0.42 | 0.002 | -0.68 | <0.001 |
| OPCs | 0.95 | 0.002 | 0.89 | 0.027 | -0.40 | 0.003 | -0.65 | <0.001 |
| Oligodendrocytes | 0 | 0.280 | 0 | 0.360 | -0.37 | 0.006 | -0.52 | <0.001 |
| Pericytes | 0.09 | 0.258 | 0.35 | 0.253 | -0.42 | 0.001 | -0.64 | <0.001 |

^1^ Expression ~ 1+ β_1_eigenvector1 + β_2_eigenvector2+ ε

^2^ Comparison to null model with 10000 permutations of spatial maps with matched spatial autocorrelation ^121^

**Table C: Model comparison for prediction of resting state functional connectivity**

|  | | |  |  | | |  | | mean squared error (mse) | | | |
| --- | --- | --- | --- | --- | --- | --- | --- | --- | --- | --- | --- | --- |
| features | | | fusion | embedding | | | learner | | HCP-style | | Freesurfer-style | |
| GD | MPC | TS |  | E1 | E2 | E3 | linear | ML | mean | std | mean | std |
| x |  |  |  |  |  |  | x |  | 0.911 | 0.109 | 0.856 | 0.167 |
|  | x |  |  |  |  |  | x |  | 0.907 | 0.114 | 0.858 | 0.164 |
|  |  | x |  |  |  |  | x |  | 0.920 | 0.046 | 0.900 | 0.050 |
| x | x | x | x |  |  |  | x |  | 1.425 | 0.286 | 1.348 | 0.494 |
| x |  |  | x | x | x |  | x |  | 0.808 | 0.150 | 3.154 | 4.017 |
|  | x |  | x | x | x |  | x |  | 1.282 | 0.344 | 6.303 | 11.129 |
|  |  | x | x | x | x |  | x |  | 0.872 | 0.087 | 1.283 | 0.704 |
| x | x |  | x | x | x |  | x |  | 2.521 | 1.842 | 0.681 | 0.214 |
| x |  | x | x | x | x |  | x |  | 0.879 | 0.161 | 0.995 | 0.418 |
|  | x | x | x | x | x |  | x |  | 0.834 | 0.136 | 5.567 | 9.878 |
| x | x | x | x | x | x |  | x |  | 0.751 | 0.179 | 0.676 | 0.216 |
| x | x | x | x | x | x | x | x |  | 0.721 | 0.173 | 0.722 | 0.241 |
| x |  |  | x | x | x |  |  | x | 0.647 | 0.145 | 1.272 | 0.433 |
|  | x |  | x | x | x |  |  | x | 1.341 | 0.332 | 1.024 | 0.357 |
|  |  | x | x | x | x |  |  | x | 0.844 | 0.094 | 1.306 | 0.326 |
| x | x |  | x | x | x |  |  | x | 1.596 | 0.427 | 0.494 | 0.188 |
| x |  | x | x | x | x |  |  | x | 0.781 | 0.142 | 0.715 | 0.210 |
|  | x | x | x | x | x |  |  | x | 0.786 | 0.142 | 1.076 | 0.393 |
| **x** | **x** | **x** | **x** | **x** | **x** |  |  | **x** | **0.492** | **0.159** | **0.484** | **0.192** |
| x | x | x | x | x | x | x |  | x | 0.514 | 0.145 | 0.436 | 0.217 |

*Note: bolded row is the primary structural manifold model.*
