# Supplementary material for: A multi-scale cortical wiring space links cellular architecture and functional dynamics in the human brain
Source: PLoS Biol. 2020 Nov 30;18(11):e3000979. doi: 10.1371/journal.pbio.3000979 (PMC7728398; doi:10.1371/journal.pbio.3000979)
Supplement: Attachment — Submitted filename: Paquola_response_R1.docx [file pbio.3000979.s015.docx]

**Response to Editors and Reviewers (PBIOLOGY-D-20-00780R1)**

We would like to thank the Editors and Reviewers for their positive evaluations, constructive comments, and for the opportunity to submit a revised manuscript. We feel that the comments and suggestions greatly improve our manuscript. We have addressed all questions and suggestions of the Editors and Reviewers in a point-by-point fashion below and highlighted the corresponding changes in the manuscript in yellow.

**Editors**

**You'll see that both reviewers are broadly positive about you study, but each raises some concerns that must be addressed before further consideration. While reviewer #1 only has some modest requests, reviewer #2 suggests a number of improvements to the methods (metrics of distance, parcellation, treatment of task vs resting-state data, etc.) and wonders whether your presentation might benefit from breaking up the Figures somewhat (we would tend to agree with the reviewers' comments on this point).**

We thank the Editors for this helpful guidance. Following the points raised by Reviewer #2, we reconstructed cortical wiring using cortex-constrained geodesic distance, using a multimodal parcellation and using a functional parcellation. We found that the structural manifold was robust to such adjustments and illustrate these results a new **Supplementary Figure S8**.

We also expanded the description of the task fMRI analysis to clarify the motivation, choice of measure and independence from the resting state analysis (P.16):

*“The procedure was repeated using functional connectivity across different tasks to demonstrate the flexibility of the wiring space in governing varied states of functional organisation, as functional connectivity derived from task fMRI exhibits both similarities and differences to resting state functional connectivity (Glasser et al., 2018). In line with previous work (Shine et al., 2019), time series from each of the seven tasks underwent a minimal pre-processing (Glasser et al., 2013) and were then averaged within node. This was followed by z-standardization and concatenation. Functional connectivity was then calculated as the inverse of Euclidean distance between these concatenated and normalized node-wise timeseries. Euclidean distance was opted for over Pearson correlation because strong, periodic BOLD fluctuations evoked by tasks produce outliers in the timeseries, and thus violate the assumptions of Pearson correlation (Horwitz, 2003; Mohanty et al., 2020). Task block structure was not regressed from the data.”*

As recommended by the Editors and Reviewer #2 we split **Figures 2** and **4** and expanded captions.

**Figure 2 | Neurobiological substrates of the wiring space. (A)** A 3D *post mortem* histological reconstruction of a human brain(Amunts et al., 2013) was used to estimate cytoarchitectural similarity and externopyramidisation. Here, we present a coronal slice, a drawing of cytoarchitecture (Vogt and Vogt, 1919), magnified view of cortical layers in BigBrain and a staining intensity profile with example of calculation of externopyramidisation (Sanides, 1962). **(B)** Matrix and density plot depict the correlation between BigBrain-derived cytoarchitectural similarity and wiring distance between pairs of regions. **(C)** Externopyramidisation, projected onto the cortical surface and into the wiring space, is highest at the bottom of the structural manifold.

**Figure 3 | Neurobiological substrates of the wiring space. (A)** mRNA-seq probes, assigned to eleven representative nodes (coloured as in **Figure 1C**, *i.e.,* their position in the wiring space), provided good coverage of the space and enabled characterisation of cell-type specific gene expression patterns. Average cell-type specific gene expression patterns projected in the wiring space, with brighter colours signifying higher expression. **(B)** Equally spaced intercardinal axes superimposed on the wiring space, and below, line plots showing correlation of gene expression patterns with each of the axes. Colours correspond to the cell-types shown in part A. **(C)** Strongest axis of variation (*i.e.,* maximum |r|) in expression of each cell-type overlaid on the structural manifold.

**Figure 5 | Large-scale organisation of coherence in the structural manifold. (A)** Intracerebral implantations of ten epileptic patients were mapped to the cortical surface, and intracortical EEG contacts selected. We studied five minutes of wakeful rest. **(B)** Mean and standard deviation in the variance explained in undirected coherence by wiring space features using adaboost machine learning across all nodes. **(C)** Clusters of the wiring space. **(D)** Phase slope index (Ψ) was calculated for each pair of intra-subject electrodes, then cluster-to-cluster estimates were derived from a linear mixed effect model. Pearson correlation across Ψ estimates was used to measure the similarity of clusters, and the major axis of regional variation was identified via principle component analysis. *i)* Average Ψ spectra for each region coloured by loading on the first principle component (accounting for 39% of variance). *ii)* Component loadings presented in the wiring space and on the cortical surface illustrate a gradient from upper left regions, corresponding to central areas, toward lower right areas, corresponding to temporal and limbic areas. *iii)* Lasso regularisation demonstrates the contribution of cell-type specific gene expression (colours matching **Figure 3**) and externopyramidisation (red) to explain variance in the component loadings. Shaded areas show the standard deviation in fitted least-squares regression coefficients across leave-one-observation-out iterations. For example, inhibitory neurons expression levels (green) are closely related to the component loading, as shown in the scatterplot.

**Figure 6 | Hierarchical information processing is organised within the structural manifold.** *Top.* The most influential frequencies on the component loading were identified through a correlation of average Ψ spectra (**Figure 5Di**) with the component loading. For the global maxima (28Hz, left) and minima (97Hz, right), we present cluster-to-cluster Ψ estimates, thresholded at p<0.05. *Below*. Suprathreshold edges are plotted as a hierarchical schema, where the directed coherence estimates indicate flow of oscillations from the top to the bottom of the hierarchy. The hierarchical level of each cluster is also presented on the cortical surface and in the wiring space, illustrating unique spatial patterns of the frequency-specific hierarchies.

**Reviewer #1:**

**Thank you for inviting me to review this manuscript by Paquola and colleagues, in which the authors conducted a sophisticated multi-modal analysis of human brain imaging data in an effort to better characterize the relationship between structure and function. The method leverages recent results from this group that have linked microstructural heterogeneity with macroscale organization. The results of this study are compelling, and extend their previous work into new domains, including more detailed micro-structural properties (from the BigBrain atlas) and high temporal resolution structural EEG data. The methods are well-motivated and described, and the statistical approaches are all appropriately justified.**

We would like to thank the Reviewer for the positive evaluation and helpful comments.

**I have a few minor points that I hope will help to improve the manuscript: Did the authors consider any other dimensionality reduction approaches? For instance, principal component analysis is similar to diffusion map embeddings, and can provide even more robust results than DME when applied to data that are well-suited to linear dimensionality reduction.**

Diffusion map embedding was selected to preserve local geometry within the overarching eigenvectors, and to also follow previous work of our group based on this technique (Paquola et al., 2019a, 2019b). In practice, the first two eigenvectors are often quite similar irrespective of whether other linear (*e.g.,* PCA) or other nonlinear (*e.g.,* Laplacian eigenmaps) approaches are used. We could confirm this in the current data (r>0.99) (P.13) and illustrate these alternatives in a new **Supplementary Figure S8**:

**Figure S8: Robustness of the structural manifold to methodological variations. A)** Illustration of the structural manifold used for the primary analyses. **B-C)** Near-identical first and second eigenvectors are produced using alternative dimensionality reduction approaches. **D-E)** The spatial pattern of the first two eigenvectors was conserved using alternative parcellations. **F**) Cortex-constrained geodesic distance, rather than geodesic distance that propagates through grey/white matter volumes, produces a hemisphere-split as the first eigenvector, however, the second and third eigenvectors are highly similar to the primary first and second eigenvectors.

*“Furthermore, the first two eigenvectors were robust across alternative dimensionality reduction approaches, such as linear principle component analysis (r>0.99) and nonlinear Laplacian eigenmaps (r>0.99).”*

**I'm not sure that I fully appreciate the authors interpretation of the 'integrative core'. From my understanding, regions that have small loadings onto the wiring space (i.e., those that exist in the middle of the plot in Figure 1D) are those that explained the least variance (at least with respect to the first two eigenvectors). It's not clear that identifying those particular regions has a specific interpretation per se. It's also not really clear precisely how the authors identified the 'integrative core'. I recommend that the authors expand on this section, and highlight precisely how the metrics were calculated in the main text.**

We thank the Reviewer for highlighting the potentially ambiguous terminology and interpretation. We have removed the term “integrative core” and instead use the average manifold distance of a node to all other nodes as a measure of increasing specialisation/distinctiveness. As recommended, we expanded the section to clarify the calculation of the metrics and provided interpretations that are closer to the observed data (P.4, P.6).

*“Nodes with high wiring affinity are close by, while dissimilar regions have a greater wiring distance from other regions. Analysing the average wiring distance of each node to all other nodes (calculated as geodesic distance in the 2D manifold), we found that primary sensory areas, such as the calcarine sulcus and superior precentral gyrus, exhibited the most distinctive, specialised cortical wiring (****Figure 1D****).”*

*“First, we mapped previously established intrinsic functional communities (Yeo et al., 2011) into the wiring space and inspected their relative wiring distances (****Figure 4A-C****). Sensory networks were located in the lower (visual) and upper (somatomotor) left extremities of the new coordinate system, concordant with their distinctive connectivity profiles and more specialised functional roles. In contrast, transmodal default, frontoparietal and limbic networks were located more towards the right extremities in the wiring space. The dorsal attention network created a frontier between sensory networks and the transmodal extremity, while the ventral attention also subsumed an intermediary position at the centre of the wiring space.”*

**Reviewer #2:**

**[identifies himself as Matthew F. Glasser M.D. Ph.D.]**

**The authors present an interesting multimodal manuscript investigating a core wiring coordinate system in the human brain.  A strength of this paper is the martialing of a very diverse and comprehensive set of modalities to compare with their model.  The paper is let down by some puzzling methodological decisions.  Also, it is a bit challenged by the very dense figures, which may be hard for some readers to parse, though I recognize that the authors are trying to cover a lot of material.  A table summarizing the MSE and R2 values for various analyses might also be beneficial.**

We would like to thank Dr Glasser for his positive appraisal of the manuscript and helpful insights on how to improve clarity. As suggested, we split Figures (*i.e.,* Figures 2 and 4) and expanded the captions. We also included Supplementary Tables summarising R^2^ and MSE values for model comparisons. We respond to the all other comments in line below.

**Table S1**: Variance explained in externopyramidisation with alternative input measures

|  | Adjusted R^2^ |
| --- | --- |
| GD only | 0.50975 |
| MPC only | 0.49381 |
| TS only | 0.62106 |
| GD + MPC | 0.64716 |
| GD + TS | 0.52581 |
| MPC + TS | 0.54945 |
| GD, MPC + TS | 0.65076 |

**Table S3: Model comparison for prediction of resting state functional connectivity**

| features | | | fusion | embedding | | | learner | | mean squared error (mse) | |
| --- | --- | --- | --- | --- | --- | --- | --- | --- | --- | --- |
| GD | MPC | TS |  | E1 | E2 | E3 | linear | ML | mean | std |
| x |  |  |  |  |  |  | x |  | 0.856 | 0.167 |
|  | x |  |  |  |  |  | x |  | 0.858 | 0.164 |
|  |  | x |  |  |  |  | x |  | 0.900 | 0.050 |
| x | x | x | x |  |  |  | x |  | 1.348 | 0.494 |
| x |  |  | x | x | x |  | x |  | 3.154 | 4.017 |
|  | x |  | x | x | x |  | x |  | 6.303 | 11.129 |
|  |  | x | x | x | x |  | x |  | 1.283 | 0.704 |
| x | x |  | x | x | x |  | x |  | 0.681 | 0.214 |
| x |  | x | x | x | x |  | x |  | 0.995 | 0.418 |
|  | x | x | x | x | x |  | x |  | 5.567 | 9.878 |
| x | x | x | x | x | x |  | x |  | 0.676 | 0.216 |
| x | x | x | x | x | x | x | x |  | 0.722 | 0.241 |
| x |  |  | x | x | x |  |  | x | 1.272 | 0.433 |
|  | x |  | x | x | x |  |  | x | 1.024 | 0.357 |
|  |  | x | x | x | x |  |  | x | 1.306 | 0.326 |
| x | x |  | x | x | x |  |  | x | 0.494 | 0.188 |
| x |  | x | x | x | x |  |  | x | 0.715 | 0.210 |
|  | x | x | x | x | x |  |  | x | 1.076 | 0.393 |
| x | x | x | x | x | x |  |  | x | 0.484 | 0.192 |
| x | x | x | x | x | x | x |  | x | 0.436 | 0.217 |

**I have the following major concerns about the methods:**
**1)  I didn't understand the rationale for the modified geodesic distance measure.  If the purpose is to measure distance across the cortical sheet, the standard approach to geodesic distance is more appropriate.  If the purpose is to measure putative axonal length, a better approach would be to actually measure distance using tractography (which can be done with FSL's probtrackx2 tool).  Given the critical importance of distance to the overall measure, it seems that the distance-based approach must be more clearly neurobiologically motivated.**

We chose to modify the geodesic distance measure for two reasons over a pure surface based approach:

(i) Propagating geodesic distance via grey and white matter voxels allows for the mapping of distances between hemispheres, unlike the cortex-constrained geodesic distance that is limited to one hemisphere. Notably, this approach also operationalized the distance mapping to be independent from diffusion MRI tractography to maximise the uniqueness of each cortical wiring feature and to avoid introducing potential biases of tractography into multiple measures.
(ii) Compared to a surface based approach, the distance propagation through a combined GM and WM mask also captures a broader range of short-range connections, including both intracortical horizontal axonal fibers as well as short fibres crossing gyri (Catani et al., 2012; Schüz et al., 2002).

*“This approach differs from previous implementations of cortex-constrained geodesic distance (Ecker et al., 2013; Hong et al., 2017; Margulies et al., 2016) by involving paths through the grey and white matter, allowing for jumps within gyri and interhemispheric projections (Larivière et al., 2020). Thereby, geodesic distance may be related to intracortical axonal fibres, as well as short-range association fibres (Catani et al., 2012).”*

A previous study (Lariviere et al. 2020) from our group has already shown the utility of this geodesic distance formulation in the context of stratifying functional connections in the epilepsies. Nevertheless, we appreciate that this is a relatively new approach in the field and examined the structural manifold using cortex-constrained geodesic distance as well. Notably the first eigenvector divides the two hemispheres because of the inability of cortex-constrained geodesic distance to cross the corpus callosum. The second and third eigenvectors are highly similar to our primary analysis, however. These findings are now presented on P.15 and in **Supplementary Figure S8** (on next page).

*“The wiring space model can be flexibly adapted to incorporate other features, or other variants of the utilized measures. One potential exploration could be to choose a geodesic distance calculation that is purely defined within the cortical grey matter, instead of the current approach that combines grey and white matter compartments. While this approach does not readily allow for the modeling of inter-hemispheric connections, it could be more specific for the approximation of horizontal fibers. Figure S8 shows a proof-of-concept example of a wiring space that incorporated cortex-wide geodesic distances.”*

**2)    Why not use the HCP's multi-modal cortical parcellation from Glasser et al., 2016 Nature or some other functionally relevant parcellation?  A random parcellation based on gyral and sulcal boundaries is unlikely to be optimal for either neurobiological interpretation or computation of functional connectivity.  I made a similar comment in the author's prior paper on myelin covariance.**

We selected a parcellation scheme based on cortical morphometry to *a)* follow our previous study (Paquola et al., 2019b) and *b)* in line with our aim to construct a wiring scheme that is primarily informed by structural neuroanatomy. The parcellation we chose also ensured *c)* that parcels were approximately equal sized. We have clarified this motivation in the methods as follows:

*“Cortical wiring features were mapped between 200 spatially contiguous cortical ‘nodes’. The parcellation scheme is a semi-random division of the Desikan Killany atlas (Desikan et al., 2006) (in line with our aim to construct a wiring scheme that is primarily informed by structural neuroanatomy) and contains approximately equal sized nodes.”*

As recommended, we repeated the structural manifold construction using a multimodal parcellation (Glasser) and a functionally defined parcellation (Schaefer 800). Surface representations of the structural manifold eigenvectors were highly similar, supporting robustness of our approach to parcellation. The findings are now detailed in the new **Supplementary Figure S8.**

*“The procedure was also validated using a 360 node multi-modal defined parcellation* (Glasser et al., 2016) *and a 800 node functional parcellation* (Schaefer et al., 2018)*, which produced similar structural manifolds to the primary analysis (0.64>r>0.80) (Figure S8).”*

**Figure S8: Robustness of the structural manifold to methodological variations. A)** Illustration of the structural manifold used for the primary analyses. **B-C)** Near-identical first and second eigenvectors are produced using alternative dimensionality reduction approaches. **D-E)** The spatial pattern of the first two eigenvectors was conserved using alternative parcellations. **F**) Cortex-constrained geodesic distance, rather than geodesic distance that propagates through grey/white matter volumes, produces a hemisphere-split as the first eigenvector, however, the second and third eigenvectors are highly similar to the primary first and second eigenvectors.

**3) The task fMRI data are used for functional connectivity, but it is not said if they were denoised or not. Also, it is unclear why the connectivity measure is changed for task functional connectivity.  Task data cannot be "made into" resting state data, so it would be better to just use the same methods that one uses with resting state data.  There are both similarities and differences between the functional networks that make up HCP resting state and task fMRI data as shown in Glasser et al., 2018 Neuroimage.**

Denoising of task fMRI was performed within the HCP minimal preprocessing pipeline (Glasser et al.2013, Neuroimage), including motion correction, EPI distortion correction and exclusion of voxels with locally high coefficient of variation in the volume-to-surface mapping. Functional connectivity was calculated as the Euclidean distance between parcel-wise timeseries because the timeseries violate the assumption of Pearson correlation on the absence of outliers. This is likely due to strong BOLD fluctuations evoked periodically by the tasks. Notably, rs-fMRI and task-fMRI were evaluated independently so as to not make comparisons between different measures of functional connectivity. Rather, the motivation of the task analysis was to demonstrate the flexibility of the structural manifold approach to describe connectivity across cognitive states. We further clarified this in the methods, as follows:

*“The procedure was repeated using functional connectivity across different tasks to demonstrate the flexibility of the wiring space in governing varied states of functional organisation, as functional connectivity derived from task fMRI exhibits both similarities and differences to resting state functional connectivity (Glasser et al., 2018). In line with previous work (Shine et al., 2019), minimally-preprocessed (Glasser et al., 2013) timeseries from each of the seven tasks were averaged within node, z-standardised, and concatenated. Functional connectivity was then calculated as the inverse of Euclidean distance between these concatenated and normalized node-wise timeseries. Euclidean distance was opted for over Pearson correlation because strong, periodic BOLD fluctuations evoked by tasks produce outliers in the timeseries, and thus violate the assumptions of Pearson correlation (Horwitz, 2003; Mohanty et al., 2020). Task block structure was not regressed from the data.”*

**Minor Concerns:**

**1) It is noted that HCP subjects were asked not to fall asleep, but it is known that many did (Glasser et al., 2018 Neuroimage).  Not sure if this affects the current study though.**

We thank the Reviewer for pointing this out and have amended the *Methods* as follows (P.12):

*“Participants were instructed to keep their eyes open, look at fixation cross, and not fall asleep. Nevertheless, some subjects were drowsy and may have fallen asleep (Glasser et al., 2018), and the group-averages investigated in the present study do not address these inter-individual differences.”*

**2) The HCP resting state and task fMRI scan length are incorrect: Resting state was a total of 3456 seconds across 4 runs and task was 2793.6 seconds across 14 runs.**

We thank the Reviewer and have corrected the acquisition lengths in the *Methods* as follows (P.11 & P.12):

*“Four rs-fMRI scans were acquired using multi-band accelerated 2D-BOLD echo-planar imaging (2mm isotropic voxels, matrix=104×90, 72 sagittal slices, TR=720ms, TE=33ms, flip angle=52°, mb factor=8, 1200 volumes/scan, 3456 seconds).”*

*“Seven task-evoked fMRI scans (working memory, gambling, motor, language, social cognition, relational processing and emotion processing (Barch et al., 2013)) were acquired with the same echo-planar imaging sequence as rs-fMRI with a total run time of 2793.6 seconds”*

**3)    "then subjected to ICA-FIX for removal of additional noise" sounds kind of pejorative.  Please rephrase.**

The Methods have been updated as follows (P.12):

*“BOLD timeseries were corrected for gradient nonlinearity, head motion, bias field and scanner drifts, then structured “noise” components were removed using ICA-FIX, further reduce the influence of motion, non-neuronal physiology, scanner artefacts and other nuisance sources* (Salimi-Khorshidi et al., 2014).*”*

**4)    The immediately following sentence should be reworded: "The data were resampled from volume to MSMAll functionally aligned surface space."**

We thank the Reviewer for the suggestion and have implemented this wording (P.12)

*“The rs-fMRI data were resampled from volume to MSMAll functionally aligned surface space* (Robinson et al., 2018, 2014)*.”*

**5) I still don't understand the oversampling of the surface profiles.  Even for 0.7mm isotropic HCP data, for a thick area at 4mm, this means an average of 0.33mm between surfaces and for a thin area at 1.6mm an average of 0.13mm between surfaces.**

We acknowledge that the number of surfaces would reflect oversampling if voxels were perfectly aligned with the vertices, however, the vertices may intersect voxels in varying degrees. The lack of alignment between voxels and vertices confers difficulty in establishing the ideal number of surfaces, as does the inter-subject and inter-regional variability. Therefore, we determined the optimal number of surfaces based on the stability of the resultant MPC matrix (Paquola et al., 2019b). This level of sampling also conforms with our prior work showing the high overlap of gradients derived from T1w/T2w and the BigBrain, sampled at 100 micron resolution (Paquola et al., 2019b). We recently further validated this approach through comparison of the profiles with hyperstained myelin pictures (Paquola et al., 2019a; Vogt and Vogt, 1919). We expanded upon this point in the methods as follows (P.12):

*“In brief, we generated 12 equivolumetric surfaces between the outer and inner cortical surfaces (Waehnert et al., 2014; Wagstyl et al., 2018), and systematically sampled T1w/T2w values along linked vertices across the whole cortex. Even though the number of surfaces exceeds the maximum number of voxels within the cortical ribbon, this slight oversampling confers high stability of the resultant microstructure profile covariance matrix (Paquola et al., 2019b)”*

**6) Please clarify that the equal volume surfaces used are from a similar implementation to that in Connectome Workbench's -surface-cortex-layer command or Waehnert et al., 2014 Neuroimage.**

Equivolumetric surface construction was operationalised using the “laminar tools” package (Wagstyl etl al., 2018) which is based on the Waehnert et al., 2014 algorithm. We added this citation on (P.12):

*“In brief, we generated 12 equivolumetric surfaces between the outer and inner cortical surfaces (Waehnert et al., 2014; Wagstyl et al., 2018),”*

**7) I didn't follow the "log-domain intensity normalization" of "fiber orientation distributions."**

We have now clarified the diffusion processing as follows (P.12):

“*Fibre orientation distributions (i.e.: the apparent density of fibres as a function of orientation) were modelled from the diffusion-weighted MRI with multi-shell multi-tissue spherical deconvolution* (Jeurissen et al., 2014)*, then values were normalised in the log domain to optimise the sum of all tissue compartments towards 1, under constraints of spatial smoothness.”*

**8) What is meant by "spike regression" in the resting state denoising.  This is not a step recommended by the HCP for their data.**

As per HCP recommendations, spike regression was not implemented for the HCP data. Spike regression was performed on the independent replication dataset. Specifically, framewise displacement was regressed from the timeseries, in line with other recommendations on optimal preprocessing to reduce the influence of head motion (Lemieux et al., 2007; Satterthwaite et al., 2013). By using alternative preprocessing strategies in each dataset, we hope to demonstrate that the specific preprocessing pipeline is not a major determinant of our findings. We have added these citations to the relevant section of the *Methods* (P.16)

*“The rs-fMRI data was additionally denoised using an in-house trained ICA-FIX classifier (Griffanti et al., 2014; Salimi-Khorshidi et al., 2014) as well as spike regression (Lemieux et al., 2007; Satterthwaite et al., 2013).”*

**9) What approach was used for surface registration in the replication data?  Folding or functional? Using FreeSurfer or MSM?**

Surface based registration was performed using Freesurfer’s spherical registration in the replication cohort. We have amended the *Methods* for clarity (P.16):

*“Timeseries were sampled on native cortical surfaces and resampled to fsaverage via surface-based registration.”*

**Response to Reviewers - References**

Amunts, K., Lepage, C., Borgeat, L., Mohlberg, H., Dickscheid, T., Rousseau, M.-E., Bludau, S., Bazin, P.-L., Lewis, L.B., Oros-Peusquens, A.-M., Shah, N.J., Lippert, T., Zilles, K., Evans, A.C., 2013. BigBrain: An Ultrahigh-Resolution 3D Human Brain Model. Science (80-. ). 340, 1472–1475. https://doi.org/10.1126/science.1235381

Barch, D.M., Burgess, G.C., Harms, M.P., Petersen, S.E., Schlaggar, B.L., Corbetta, M., Glasser, M.F., Curtiss, S., Dixit, S., Feldt, C., Nolan, D., Bryant, E., Hartley, T., Footer, O., Bjork, J.M., Poldrack, R., Smith, S., Johansen-Berg, H., Snyder, A.Z., Van Essen, D.C., WU-Minn HCP Consortium, 2013. Function in the human connectome: task-fMRI and individual differences in behavior. Neuroimage 80, 169–89. https://doi.org/10.1016/j.neuroimage.2013.05.033

Catani, M., Dell’Acqua, F., Vergani, F., Malik, F., Hodge, H., Roy, P., Valabregue, R., Thiebaut de Schotten, M., 2012. Short frontal lobe connections of the human brain. Cortex 48, 273–291. https://doi.org/10.1016/j.cortex.2011.12.001

Desikan, R.S., Segonne, F., Fischl, B., Quinn, B.T., Dickerson, B.C., Blacker, D., Buckner, R.L., Dale, A.M., Maguire, R.P., Hyman, B.T., Albert, M.S., Killiany, R.J., Ségonne, F., Fischl, B., Quinn, B.T., Dickerson, B.C., Blacker, D., Buckner, R.L., Dale, A.M., Maguire, R.P., Hyman, B.T., Albert, M.S., Killiany, R.J., 2006. An automated labeling system for subdividing the human cerebral cortex on MRI scans into gyral based regions of interest. Neuroimage 31, 968–980. https://doi.org/10.1016/j.neuroimage.2006.01.021

Ecker, C., Ronan, L., Feng, Y., Daly, E., Murphy, C., Ginestet, C.E., Brammer, M., Fletcher, P.C., Bullmore, E.T., Suckling, J., Baron-Cohen, S., Williams, S., Loth, E., Murphy, D.G.M., Bailey, A.J., Baron-Cohen, S., Bolton, P.F., Bullmore, E.T., Carrington, S., Chakrabarti, B., Daly, E.M., Deoni, S.C., Ecker, C., Happe, F., Henty, J., Jezzard, P., Johnston, P., Jones, D.K., Lai, M.C., Lombardo, M. V., Madden, A., Mullins, D., Murphy, C.M., Murphy, D.G., Pasco, G., Sadek, S., Spain, D., Steward, R., Suckling, J., Wheelwright, S., Williams, S.C., 2013. Intrinsic gray-matter connectivity of the brain in adults with autism spectrum disorder. Proc. Natl. Acad. Sci. 110, 13222–13227. https://doi.org/10.1073/pnas.1221880110

Glasser, M.F., Coalson, T.S., Bijsterbosch, J.D., Harrison, S.J., Harms, M.P., Anticevic, A., Van Essen, D.C., Smith, S.M., 2018. Using temporal ICA to selectively remove global noise while preserving global signal in functional MRI data. Neuroimage 181, 692–717. https://doi.org/10.1016/j.neuroimage.2018.04.076

Glasser, M.F., Coalson, T.S., Robinson, E.C., Hacker, C.D., Harwell, J., Yacoub, E., Ugurbil, K., Andersson, J., Beckmann, C.F., Jenkinson, M., Smith, S.M., Van Essen, D.C., 2016. A multi-modal parcellation of human cerebral cortex. Nature 536, 171–178. https://doi.org/10.1038/nature18933

Glasser, M.F., Sotiropoulos, S.N., Wilson, J.A., Coalson, T.S., Fischl, B., Andersson, J.L., Xu, J., Jbabdi, S., Webster, M., Polimeni, J.R., Van Essen, D.C., Jenkinson, M., 2013. The minimal preprocessing pipelines for the Human Connectome Project. Neuroimage 80, 105–124. https://doi.org/10.1016/J.NEUROIMAGE.2013.04.127

Griffanti, L., Salimi-Khorshidi, G., Beckmann, C.F., Auerbach, E.J., Douaud, G., Sexton, C.E., Zsoldos, E., Ebmeier, K.P., Filippini, N., Mackay, C.E., Moeller, S., Xu, J., Yacoub, E., Baselli, G., Ugurbil, K., Miller, K.L., Smith, S.M., 2014. ICA-based artefact removal and accelerated fMRI acquisition for improved resting state network imaging. Neuroimage 95, 232–247. https://doi.org/10.1016/j.neuroimage.2014.03.034

Hong, S.-J., Valk, S.L., Di Martino, A., Milham, M.P., Bernhardt, B.C., 2017. Multidimensional Neuroanatomical Subtyping of Autism Spectrum Disorder. Cereb. Cortex 1–11. https://doi.org/10.1093/cercor/bhx229

Horwitz, B., 2003. The elusive concept of brain connectivity. Neuroimage 19, 466–470. https://doi.org/10.1016/S1053-8119(03)00112-5

Jeurissen, B., Tournier, J.-D., Dhollander, T., Connelly, A., Sijbers, J., 2014. Multi-tissue constrained spherical deconvolution for improved analysis of multi-shell diffusion MRI data. Neuroimage 103, 411–426. https://doi.org/10.1016/J.NEUROIMAGE.2014.07.061

Larivière, S., Weng, Y., Vos de Wael, R., Royer, J., Frauscher, B., Wang, Z., Bernasconi, A., Bernasconi, N., Schrader, D. V., Zhang, Z., Bernhardt, B.C., 2020. Functional connectome contractions in temporal lobe epilepsy: Microstructural underpinnings and predictors of surgical outcome. Epilepsia 61, 1221–1233. https://doi.org/10.1111/epi.16540

Lemieux, L., Salek-Haddadi, A., Lund, T.E., Laufs, H., Carmichael, D., 2007. Modelling large motion events in fMRI studies of patients with epilepsy. Magn. Reson. Imaging 25, 894–901. https://doi.org/10.1016/j.mri.2007.03.009

Margulies, D.S., Ghosh, S.S., Goulas, A., Falkiewicz, M., Huntenburg, J.M., Langs, G., Bezgin, G., Eickhoff, S.B., Castellanos, F.X., Petrides, M., Jefferies, E., Smallwood, J., 2016. Situating the default-mode network along a principal gradient of macroscale cortical organization. Proc. Natl. Acad. Sci. 113, 12574–12579. https://doi.org/10.1073/pnas.1608282113

Mohanty, R., Sethares, W.A., Nair, V.A., Prabhakaran, V., 2020. Rethinking Measures of Functional Connectivity via Feature Extraction. Sci. Rep. 10. https://doi.org/10.1038/s41598-020-57915-w

Paquola, C., Bethlehem, R.A., Seidlitz, J., Wagstyl, K., Romero-Garcia, R., Whitaker, K.J., Vos de Wael, R., Williams, G.B., Vértes, P.E., Margulies, D.S., Bernhardt, B., Bullmore, E.T., 2019a. Shifts in myeloarchitecture characterise adolescent development of cortical gradients. Elife 8. https://doi.org/10.7554/eLife.50482

Paquola, C., Vos De Wael, R., Wagstyl, K., Bethlehem, R.A.I., Hong, S.-J., Seidlitz, J., Bullmore, E.T., Evans, A.C., Misic, B., Margulies, D.S., Smallwood, J., Bernhardt, B.C., 2019b. Microstructural and functional gradients are increasingly dissociated in transmodal cortices. PLOS Biol. 17, e3000284. https://doi.org/10.1371/journal.pbio.3000284

Robinson, E.C., Garcia, K., Glasser, M.F., Chen, Z., Coalson, T.S., Makropoulos, A., Bozek, J., Wright, R., Schuh, A., Webster, M., Hutter, J., Price, A., Cordero Grande, L., Hughes, E., Tusor, N., Bayly, P. V., Van Essen, D.C., Smith, S.M., Edwards, A.D., Hajnal, J., Jenkinson, M., Glocker, B., Rueckert, D., 2018. Multimodal surface matching with higher-order smoothness constraints. Neuroimage 167, 453–465. https://doi.org/10.1016/j.neuroimage.2017.10.037

Robinson, E.C., Jbabdi, S., Glasser, M.F., Andersson, J., Burgess, G.C., Harms, M.P., Smith, S.M., Van Essen, D.C., Jenkinson, M., 2014. MSM: A new flexible framework for multimodal surface matching. Neuroimage 100, 414–426. https://doi.org/10.1016/j.neuroimage.2014.05.069

Salimi-Khorshidi, G., Douaud, G., Beckmann, C.F., Glasser, M.F., Griffanti, L., Smith, S.M., 2014. Automatic denoising of functional MRI data: Combining independent component analysis and hierarchical fusion of classifiers. Neuroimage 90, 449–468. https://doi.org/10.1016/j.neuroimage.2013.11.046

Sanides, F., 1962. Die Architektonik des menschlichen Stirnhirns zugleich eine Darstellung der Prinzipien seiner Gestaltung als Spiegel der stammgeschichtlichen Differenzierung der Grosshirnrinde. Springer, Berlin.

Satterthwaite, T.D., Elliott, M.A., Gerraty, R.T., Ruparel, K., Loughead, J., Calkins, M.E., Eickhoff, S.B., Hakonarson, H., Gur, R.C., Gur, R.E., Wolf, D.H., 2013. An improved framework for confound regression and filtering for control of motion artifact in the preprocessing of resting-state functional connectivity data. Neuroimage 64, 240–256. https://doi.org/10.1016/j.neuroimage.2012.08.052

Schaefer, A., Kong, R., Gordon, E.M., Laumann, T.O., Zuo, X.-N., Holmes, A.J., Eickhoff, S.B., Yeo, B.T.T., 2018. Local-Global Parcellation of the Human Cerebral Cortex from Intrinsic Functional Connectivity MRI. Cereb. Cortex 28, 3095–3114. https://doi.org/10.1093/cercor/bhx179

Schüz, A., Braitenberg, V., Braitenberg, V., 2002. The Human Cortical White Matter: Quantitative Aspects of Cortico-Cortical Long-Range Connectivity 389–398. https://doi.org/10.1201/9780203299296-24

Shine, J.M., Breakspear, M., Bell, P.T., Ehgoetz Martens, K., Shine, R., Koyejo, O., Sporns, O., Poldrack, R.A., 2019. Human cognition involves the dynamic integration of neural activity and neuromodulatory systems. Nat. Neurosci. 22, 289–296. https://doi.org/10.1038/s41593-018-0312-0

Vogt, C., Vogt, O., 1919. Allgemeinere Ergebnisse unserer Hirnforschung. J. für Psychol. und Neurol. 25, 279–461.

Waehnert, M.D., Dinse, J., Weiss, M., Streicher, M.N., Waehnert, P., Geyer, S., Turner, R., Bazin, P.-L., 2014. Anatomically motivated modeling of cortical laminae. Neuroimage 93, 210–220. https://doi.org/10.1016/J.NEUROIMAGE.2013.03.078

Wagstyl, K., Paquola, C., Bethlehem, R., Huth, A., 2018. kwagstyl/surface_tools: Initial release of equivolumetric surfaces. https://doi.org/10.5281/ZENODO.1412054

Yeo, B.T.T., Krienen, F.M., Sepulcre, J., Thomas Yeo, B.T., Krienen, F.M., Sepulcre, J., Sabuncu, M.R., Lashkari, D., Hollinshead, M., Roffman, J.L., Smoller, J.W., Zöllei, L., Polimeni, J.R., Fischl, B., Liu, H., Buckner, R.L., Yeo, B.T.T., Krienen, F.M., Sepulcre, J., 2011. The organization of the human cerebral cortex estimated by intrinsic functional connectivity. J. Neurophysiol. 106, 1125–1165. https://doi.org/10.1152/jn.00338.2011
