# Supplementary material for: A multi-scale cortical wiring space links cellular architecture and functional dynamics in the human brain
Source: PLoS Biol. 2020 Nov 30;18(11):e3000979. doi: 10.1371/journal.pbio.3000979 (PMC7728398; doi:10.1371/journal.pbio.3000979)
Supplement: Attachment — Submitted filename: Paquola_ManiManu_response_R2_BB.docx [file pbio.3000979.s016.docx]

**Response to Editors and Reviewers (PBIOLOGY-D-20-00780R2)**

We would like to thank the Editors and Reviewers for the opportunity to revise the manuscript. We have adapted the manuscript according to each suggestion of Reviewer 2. The suggestions were constructive and improved the paper, and we appreciate that the Reviewer pointed out a potential path on how we can optimally address them. Specifically, we followed the Reviewer’s suggestions and i) removed task-based findings, ii) base our main findings on the HCP parcellation, iii) used a cortex-constrained geodesic distance metric for the main analyses. Detailed answers can be found in a point-by-point fashion below, and we highlighted the corresponding changes in the manuscript in yellow.

**Reviewer #2: [identifies himself as Matthew F. Glasser]**

The authors have incompletely addressed my concerns.  I continue to have the following major concerns, and suggest paths to resolving them that hopefully will be acceptable to the authors:

1) This statement in the response to reviews: "Denoising of task fMRI was performed within the HCP minimal preprocessing pipeline (Glasser et al.2013, Neuroimage), including motion correction, EPI distortion correction and exclusion of voxels with locally high coefficient of variation in the volume-to-surface mapping." really concerns me.  The HCP's minimal preprocessing pipelines are designed to remove spatial distortions, realign the data within and across modalities, and to exclude voxels that are likely to contain little usable signal due to corruption from blood vessels or dura.  They do not do ANY temporal denoising.  The HCP's recommended approach to temporal denoising includes at a minimum spatial ICA-based cleanup (such as that provided by the sICA+FIX pipeline that was run on the resting state data).  Unfortunately, such cleanup has not yet been released for the HCP's task fMRI data (but will be in the future); however, it is absolutely critical for a connectivity-based analysis of fMRI data.  When looking at properly cleaned HCP data (both resting state and task) there is no increase in outliers in task data over resting state (indeed the kurtosis of resting state data is slightly higher than task data).  Thus, the authors are likely misattributing the effects of strong structured artifacts that have not been removed from the task fMRI data to the task condition itself, which is inaccurate.  I don't think the task analysis is critical to the paper, so if it cannot be easily done correctly, perhaps it is best removed/saved for when properly denoised task fMRI data is available.

We agree with the Reviewer that the task analysis is not critical to the paper and should be addressed in a more comprehensive manner in a separate paper. As such, we have removed the task analyses from the manuscript and Figures as recommended.

2) I appreciate the authors including the analyses from the HCP parcellation and Schaefer parcellation. They do seem both to be quite a bit different from the gyral and sulcal parcellation results and more similar to each other.  This is expected, given that both have significant contributions from resting state functional connectivity, which has much more neurobiological relevance than folding patterns.  Thus, it seems the main analyses of the paper should be based around one or both of these and not the outlier folding parcellation.  It is also not clear to me what neurobiological property of brain organization the authors are referring to when they say "structural neuroanatomy."  It isn't one of the 4 cortical properties that have been used to define cortical areas either classically or in modern maps (i.e. cortical microstructural architecture, cortical function as measured invasively or non-invasively, connectivity—functional, diffusion based, or invasive tracer based, or topographic maps).

In line with the Reviewer’s suggestion, we adopted the multimodal HCP parcellation for the primary analyses and show these results in the main manuscript. All key findings were maintained and as such the *Discussion* has not been altered. We note two note changes in the fMRI-based *Results* that did not impact the conclusions, however:

Previously: *Further expanding the wiring space to three eigenvectors/dimensions mildly decreased the average mean squared error, but resulted in larger variance across nodes and poorer predictive accuracy at the lower limits for the prediction of resting-state functional connectivity (mean squared error=0.44±0.21; R^2^=0.56±0.22).*

Updated version: *Further expanding the wiring space to three eigenvectors/dimensions did not improve model performance (mean squared error=0.51±0.15; R^2^=0.56±0.22,* ***Table S3****).*

Previously: *Sensory networks were located in the lower (visual) and upper (somatomotor) left extremities of the new coordinate system, concordant with their distinctive connectivity profiles and more specialised functional roles. In contrast, transmodal default, frontoparietal and limbic networks were located more towards the right extremities in the wiring space. The dorsal attention network created a frontier between sensory networks and the transmodal extremity, while the ventral attention also subsumed an intermediary position at the centre of the wiring space. Together, these analyses demonstrate how the relative positioning of functional communities in the structural wiring space underpins coordination within and between functional networks.*

Updated version: *Sensory networks were primarily located in the lower (visual) and upper (somatomotor) left extremities of the new coordinate system, concordant with their distinctive connectivity profiles and more specialised functional roles. In contrast, transmodal default, frontoparietal and limbic networks were located more towards the right extremities in the wiring space. Dorsal and ventral attention networks subsumed intermediary position in the wiring space. Together, these analyses demonstrate how the relative positioning of these communities in the structural wiring space relates to the segregation of functional networks.*

The folding-based parcellation is now represented in the Supplementary Materials. We updated all Figures, Supplementary Figures, and statistics to reflect the prioritisation of the multimodal HCP parcellation. We also expanded upon the choice of parcellation schemes in the *Methods* (P.9):

*“Cortical wiring features were mapped between spatially contiguous cortical ‘nodes’. We advanced two approaches throughout all analyses, one based on multimodal parcellations recommended by HCP (“HCP-style”) and one based on sulco-gyral parcellations implemented in FreeSurfer (“FreeSurfer-style”). For HCP-style, a 360 node parcellation scheme was used, in which areal boundaries were defined by gradients of change in mean T1w/T2w intensity, cortical thickness, task-related BOLD activation and/or resting-state functional connectivity ^101^. For Freesurfer-style, a 200 node, semi-random division of the Desikan Killany atlas ^102^ was used, with approximately equal sized nodes. By providing both approaches we aim to demonstrate that our hypotheses may be supported by either style, although we did not set out to compare their performance.”*

3) The question of the correct distance metric is still unclear to me.  I think the authors are intending their distance metric to represent "distance along an axon connecting two brain areas" and not "distance along the surface."  Their inclusion of pure geodesic distance does illustrate that geodesic distances are largely driving the current results.  Although the authors are correct that the optimal approach to deriving tractography connection weights has not been found, tractography algorithms are reasonably good at measuring the physical distance between grey matter points along the most probable connection path (e.g. Donahue et al 2016 Journal of Neuroscience).  Without experimental evidence, I don't have confidence the authors' analyses would not be meaningfully different if they used estimated axonal distances.  If they don't wish to do these analyses, they need to better justify why along axonal distances are not the correct measure for their analysis of brain "wiring."

Grey/white matter constrained geodesic distance was devised to capture spatial proximity. We appreciate further empirical evidence is necessary to validate the relationship of spatial proximity to axonal distances, however. As such, we have changed the primary analyses to use cortex-constrained geodesic distance, in line with HCP recommendations and the Reviewer’s suggestion. The original geodesic distance procedure is reported in the Supplementary Materials as part of the Freesurfer-style pipeline. All key findings were maintained, showing that cortex-constrained and volume-based geodesic distance offer two alternative measurements of spatial proximity. This will inform future research to determine an optimal definition and quantification of spatial proximity for cortico-cortico wiring. The *Methods* were updated (P.9):

*“Geodesic distance (GD) was calculated across subject-specific mid-cortical surface maps in native space. Exemplar vertices of each node were defined for each subject as the vertex with minimum Euclidian distance to the subject-specific node centroid. For HCP-style, we used workbench commands to calculate the geodesic distance between exemplar vertices by travelling along the cortical midsurface ^26,27,34^. To compensate for the inability of such an approach to cross hemispheres, we mirrored the geodesic distance. For FreeSurfer-style, we adopted a recently published GD calculation approach that combines surface and volume-based geodesic distance calculations ^103^ . For each node, we matched the exemplar vertex to the nearest voxel in volumetric space, then used a Chamfer propagation (imGeodesics Toolbox;* [*https://github.com/mattools/matImage/wiki/imGeodesics*](https://github.com/mattools/matImage/wiki/imGeodesics)*) to calculate the distance to all other voxels travelling through a grey/white matter mask. This approach differs from cortex-constrained geodesic distance implemented in workbench ^26,27,34^ by involving paths through the grey and white matter, allowing for jumps within gyri and interhemispheric projections ^103^ and thereby potentially tracking intracortical, as well as short-range association fibres ^104^. We projected GD estimations back from volumetric to surface space, averaged within node and produced a symmetric GD matrix.”.*

Minor Comment:

1) Describe the replication analysis surface registration as "Timeseries were sampled on native cortical surfaces and resampled to fsaverage via folding-based FreeSurfer surface registration."

We have made the recommended update in the *Methods* [P.13].

*“Timeseries were sampled on native cortical surfaces and resampled to fsaverage via folding-based FreeSurfer surface registration* ^96^*.”*
