# Supplementary material for: A multi-scale cortical wiring space links cellular architecture and functional dynamics in the human brain
Source: PLoS Biol. 2020 Nov 30;18(11):e3000979. doi: 10.1371/journal.pbio.3000979 (PMC7728398; doi:10.1371/journal.pbio.3000979)
Supplement: Attachment — Submitted filename: PBIOLOGY-D-20-00780 PLOS Formatting Requests.docx [file pbio.3000979.s017.docx]

PLOS Formatting Requests

# For: “A multi-scale cortical wiring space links cellular architecture and functional dynamics in the human brain”

To access your manuscript and complete these changes, please follow the link in the email and find your manuscript in the ‘Submissions Needing Revision’ folder on your main menu. Please also provide a tracked changes file indicating any updates.

**This is your FINAL opportunity to make amendments to the scientific content of your manuscript.** We strongly recommend that all authors double check ALL files prior to the final versions being submitted. Content changes requested at later stages WILL NOT BE ACCEPTED.

**Questions?** Email [plosbiology@plos.org](mailto:plosbiology@plos.org) quoting your manuscript number. We also recommend reading our [author submission guidelines.](http://journals.plos.org/plosbiology/s/submission-guidelines)

# METADATA CHECK

Please ensure that all information entered in the submission form is correct. This information will be indexed in PubMed and published by *PLOS Biology* as written.

☑︎ An **Early Version** of your manuscript:

- WILL be published online ahead of the final version

If you (or your institution) are planning a press release for this article, please contact us as soon as possible to ensure that an early version of your paper is not published.

☑︎ Please check the **authorship and affiliations** list on the title page of your manuscript. These details will be used to index the authorship of your manuscript and therefore should be checked carefully for any errors.

- Please ensure that if you have any protocols associated with [protocols.io](https://www.protocols.io/) in your manuscript, they are made public before publication to guarantee immediate access to the methodological details.

**Authors: Not applicable**

Please confirm that the following elements are correct and written in full sentences. Please update as needed.

☑︎**Financial Disclosure**

☑︎**Competing Interests**

☑︎**Data Availability Statement**

**Authors: We verified and expanded these these three statements.**

# MANUSCRIPT REQUIREMENTS

Please update your manuscript with the following changes:

1. Your co-author, Raul Rodriguez Cruces ([raul.rodriguezcruces@mcgill.ca](mailto:raul.rodriguezcruces@mcgill.ca)), has not confirmed authorship of the manuscript. We have resent them the authorship confirmation email; however please check that the above email address for them is correct and follow up personally to ensure they confirm. Please note that we cannot pass your manuscript to Production until we have received confirmations from all co-authors.

**Authors: The response is pasted below:**
Begin forwarded message:

**From:** Raúl rc <raulrcruces@gmail.com>

**Subject: Re: PLOS Biology: Confirmation of added/removed author(s) for PBIOLOGY-D-20-00780R3 - [EMID:c3d5f47a38a48ea4]**

**Date:** October 12, 2020 at 9:46:51 PM EDT

**To:** Boris Bernhardt <bernhardt@cbs.mpg.de>

Yes, I approve.

On Mon, Oct 12, 2020, 6:54 AM Boris Bernhardt <[bernhardt@cbs.mpg.de](mailto:bernhardt@cbs.mpg.de)> wrote:

Hi Raul - I will need your approval of being added to Casey’s paper. Please reply with 'I approve’ to this email if you do at your earliest convenience.

Thanks

Boris

Begin forwarded message:

**From:** "PLOS Biology" <[em@editorialmanager.com](mailto:em@editorialmanager.com)>

**Subject: PLOS Biology: Confirmation of added/removed author(s) for PBIOLOGY-D-20-00780R3 - [EMID:c3d5f47a38a48ea4]**

**Date:** October 5, 2020 at 3:47:40 AM GMT-4

**To:** "Boris C Bernhardt" <[boris.bernhardt@mcgill.ca](mailto:boris.bernhardt@mcgill.ca)>

**Reply-To:** "PLOS Biology" <[plosbiology@plos.org](mailto:plosbiology@plos.org)>

CC: "Raul Rodriguez Cruces" [raul.rodriguezcruces@mcgill.ca](mailto:raul.rodriguezcruces@mcgill.ca)

2. Please provide a detailed Financial Disclosure statement. This is published with the article, therefore should be completed in full sentences and contain the exact wording you wish to be published.

   1. Please clarify all sources of funding (financial or material support) for your study. List the grants (with grant number) or organizations (with url) that supported your study, including funding received from your institution.
   2. State the initials, alongside each funding source, of each author to receive each grant.
   3. State what role the funders took in the study. If the funders had no role in your study, please state: “The funders had no role in study design, data collection and analysis, decision to publish, or preparation of the manuscript.”

4. If any authors received a salary from any of your funders, please state which authors and which funders.

If you did not receive any funding for this study, please state: “The authors received no specific funding for this work.

**Authors: Done!**

3. In your Data Availability Statement, please ensure you have provided all necessary access information in complete sentences. This statement will appear on the published version of your manuscript.

**Authors: Done!**

4. Please ensure that all authors have at least one affiliation listed on the title page and that all listed affiliations are attributed to at least one author. Affiliations should be listed in ascending numerical order in the affiliation list and should match what is provided in the Authors Information in the submission form.

   Affiliations should include an institution, a city, and a country (no postcode). US affiliations need the state as well, written out in full (with no abbreviations).

**Authors: Verified!**

5. Please ensure that all author names are spelled correctly in the manuscript file and the submission form, including the use of a middle name/initials, and any special characters (accents, umlauts, etc.). Please place first/given names before last/family names. We ask that you ensure they match verbatim. Please be aware that correct spelling and formatting of author names in the article file is vital for accurate indexing in PubMed. The following author names do not match verbatim across the article file and the submission form: Richard A. I. Bethlehem, Sara Larivière, Reinder Vos de Wael and Jeffery A. Hall

**Authors: The naming in the article file is correct; Family names are underlined.**

6. Please ensure that your Ethics Statement is available in the Methods section of your manuscript in its entirety, under the subheading “Ethics Statement”.

**Authors: The Ethics statement is now provided in the Methods, and we added the local REB number.**

7. We have noted that you have the following present in your manuscript: Data Availability Statement

   We will remove these section from your manuscript file as this information is automatically pulled from the submission form. Please make sure that the information in the submission form is correct.

**Authors: Ok!**

8. Please remove the funding information from the Acknowledgments section of your manuscript file. The published version is pulled automatically from the “Financial Disclosure” field in our submission form and must not be duplicated. Please therefore take this opportunity to check that the information in this field is correct and completed in full.

**Authors: Ok!**

9. Please make sure all references are cited in ascending numerical order in the text.

**Authors: Verified!**

10. Please upload your Supporting Information items individually, rather than in a single file, as you refer to them individually in the text. If you would like to retain the single file please rename internal items (e.g. tables) as A, B, C etc. Please also provide a legend for each under the heading Supporting Information. Supporting Information files should be less than 10 MB each. Please edit any citation of internal items within your Supporting Information files to using letters rather than numbers, e.g. “Table A in S1 Text”. Please ensure these changes are made throughout the manuscript and within the Supporting Information files themselves.

**Authors: Ok!**

11. Please ensure that all Supporting Information files use the following format verbatim throughout the manuscript (including legends and all in-text citations): S1 Fig, S1 Table, S1 Text, S1 Data, etc. Each file type should start at 1 and increase numerically. Please note, however, that the file names themselves must use an underscore rather than a space, i.e. S1_Fig.tif, S1_Table.xlsx, S1_Text.doc, S1_Data.xlsx, S1_PRISMA_Checklist.docx, etc.

**Authors: Verified!**

12. Please ensure that each supporting information file has a legend at the end of the manuscript file, after the Reference list.

**Authors: Added!**

13. Please note that your submission cannot contain any references to generic "supporting information" files and instead must refer to specific files using our naming convention (S1 Fig, S1 Table, S1 Text, S1 Data, etc.)

**Authors: verifed!**

**Before resubmitting your paper, please ensure that all files referenced in the manuscript are present in the File Inventory, as missing files will result in further delays. Please also ensure that all file names adhere to our naming conventions and that all in-text citations and supporting information file legends/captions do as well.**

If Supporting Information files are included with your article, note that these are not copyedited and will be published as they are submitted. Please ensure that these files are legible and of high quality (at least 300 dpi) in an easily accessible file format. For this reason, please be aware that any references listed in an SI file will not be indexed. For more information, see our [Supporting Information guidelines](http://journals.plos.org/plosbiology/s/supporting-information).

# FIGURE REQUIREMENTS

PLOS offers a figure-checking tool, PACE (<http://pacev2.apexcovantage.com/>), to help authors to ensure all figures meet PLOS requirements so that the quality of published figures is as high as possible. **Please use this tool to help you address the requests below.**

PACE is a digital diagnostic and conversion tool for figure files. It will provide information about any failed check(s) and, if able, will automatically convert the figure file into an acceptable file that passes quality checks. PACE requires you to register for an account to ensure your figure files are processed securely.

Please complete the following instructions regarding your figure files:

1. Please ensure that all main figures are referenced in the manuscript as Fig 1, Fig 2, etc. (including capitalization), rather than Figure 1, Figure 2, etc. Please note, however, that the file names themselves must not include the space, i.e. Fig1.tif, Fig2.tif. Please ensure that all main figure files are clearly labeled as such in the File Inventory.

**Authors: Verified!**

2. Please confirm that you either created the following figure panels, or that you will replace them with an open source alternative: brain graphics in Figs 1—6 and supplementary figs S1—S11 and S13

**Authors: All figures were created by CP!**

Here are a few open source image websites you may wish to use:
- <https://commons.wikimedia.org>
- <http://www.publicdomainpictures.net/>
- <https://openclipart.org/>

3. Please note that your figures do not currently comply with our publication requirements. For further instructions to assist with your Figure revisions, please refer to our Figure Guidelines at: <http://journals.plos.org/plosbiology/s/figures>.

   If you need assistance, please contact us directly at [graphics@jjeditorial.com](mailto:graphics@jjeditorial.com) so we can further help with this matter. Please send any relevant source files and tell us what application(s) and what operating system you are using.

   -Figures 1-6 have been submitted in a layered TIF file format which we don’t accept for Figures. To ensure that your Figures publish properly, please flatten the layers in each Figure file (Layer→Flatten Image in Photoshop, Image→Flatten Image in GIMP) and re-upload. Make sure to save your TIF files with LZW Compression turned on.

   -Figures 1-6 exceed the dimension limits for Figures. Figures must be less than 19.05cm in width, and less than 22.225cm in height. Please note that figures will be typeset at the dimensions provided. Please make the necessary adjustments and re-upload your Figures. In Photoshop: Image→Image Size. Make sure the Resample Image checkbox is NOT checked. If the Width is over 19.05cm, type 19.05 in the Width box (19.05cm is our maximum allowable width for figures). In GIMP: Image→Scale Image. Set the units of measurement, in the pull down menu next to Height, to millimeters. If the Width is over 190.5mm, type 190.5 in the Width box (19.05cm is our maximum allowable width for figures) and hit Tab. The new Height of the figure will appear, scaled proportionately to the change in Width.

   -While revising figures 1-6, please keep in mind that we ask that all text be between 8-12 point font (2.8 - 4.2 mm). It is still possible to read 5-6 point type but should only be used sparingly in your figure. For example, it is acceptable for numbers along an axis of a graph or for subscripts and superscripts. 4 point, or smaller, text is not allowed. We recommend that you print figures out to get a better idea of the text legibility.

   -Figures 1-6 are low resolution and will appear blurry or pixelated if published. Text and images should appear crisp and clear. Please note that this problem will not be detected or corrected by the PACE tool, and this will not be fixed by simply increasing the resolution of your Figures in the image editing software. Please carefully review the PLoS Figure Guidelines and provide Figure files that were originally created at 300 pixels/inch (dpi) and at least 6.68cm wide, but less than 19.05cm and under 22.225cm tall. For further information, please see the Figure Guidelines (http://journals.plos.org/plosbiology/s/figures).

   1) Export a PDF from PowerPoint/Word/Excel (or other image source file). On a Mac, click on the 'PDF' button in the printer dialog box and select 'Save as PDF'. On a PC, send the file to print and select 'Adobe PDF' or 'PDFCreator' (it's freeware) from your list of available printers.
   If you are using Adobe PDF, then go to Printer Properties, Default Settings and select Press Quality.
   If you are using PDFCreator, Go to 'Printer Properties' and then click on 'Advanced' on the bottom right. Under 'Graphic', go to 'Print Quality' and select 600dpi.
   2) PHOTOSHOP: When you open up the PDF, make sure that the 'Constrain Proportions' box is checked. Set the 'Resolution' to 300dpi (118.11 pixels/cm) and set the 'Bit Depth' to 8 bit. Use the crop tool (next to the eyedropper) to remove unnecessary white space. Go to - Image - Image Size. Make sure the 'Constrain Proportions' and 'Resample Image” boxes are checked.
   To ensure that your Figures publish properly, please delete the alpha channels from each Figure file. In Photoshop, select it in the Channels Palette and click on the trash can icon. When you save the TIFFs make sure that the 'LZW' button is checked and 'Discard Layers and Save a Copy' button is checked.
   3) GIMP: When you open up the PDF, set the resolution to 300. Make sure the chain link icon is joined together. Then change the size of your figure according to the pixel specifications mentioned in step 2) above. To ensure that your Figures publish properly, please delete the alpha channels from each Figure file. In GIMP, go to Layers - Transparency - Remove Alpha Channel. When you are done, 'SAVE AS' (or Export if GIMP 2.8). Select File Type - 1st TIFF option. Make sure to use LZW compression.
   4) Print out your figures on a desktop printer. Text and graphics should appear clear and crisp with no pixelated or blurred edges.
   5) If the resulting figures are still blurry, then your original source images are too low of a resolution. Please increase the resolution of your original source images, and repeat this process.

**Authors: All figures are saved as tiff in the appropriate resolution. We used the PACE tool for verification.**

Please note that this is your only chance to adjust figures, changes to figures (including resizing) at proof will not be accepted.

For a full outline, please see our [figure](http://journals.plos.org/plosbiology/s/figures) and [table](http://journals.plos.org/plosbiology/s/tables) preparation guidelines.

You can also upload a Striking Image to accompany your article if one is available. If this image is judged to be suitable, it may be featured on our website. Images should be high resolution, eye-catching, single panel images. If your image is from someone other than yourself, please ensure that the artist has read and agreed to the terms and conditions of the Creative Commons Attribution License. Note: we cannot publish copyrighted images.
